# Inter-hemispheric functional dysconnectivity mediates the association of corpus callosum degeneration with memory impairment in AD and amnestic MCI

**DOI:** 10.1038/srep32573

**Published:** 2016-09-01

**Authors:** Yingwei Qiu, Siwei Liu, Saima Hilal, Yng Miin Loke, Mohammad Kamran Ikram, Xin Xu, Boon Yeow Tan, Narayanaswamy Venketasubramanian, Christopher Li-Hsian Chen, Juan Zhou

**Affiliations:** 1 Center for Cognitive Neuroscience, Neuroscience and Behavioral Disorders Program, Duke-National University of Singapore Graduate Medical School, Singapore; 2 Department of Pharmacology, National University Health System, Clinical Research Centre, Singapore; 3 Memory Aging & Cognition Centre, National University Health System, Singapore; 4 Duke-NUS Graduate Medical School, National University of Singapore, Singapore; 5 St. Luke’s Hospital, Singapore; 6 Raffles Neuroscience Centre, Raffles Hospital, Singapore; 7 Clinical Imaging Research Centre, the Agency for Science, Technology and Research and National University of Singapore, Singapore

## Abstract

Evidences suggested that both corpus callosum (CC) degeneration and alternations of homotopic inter-hemispheric functional connectivity (FC) are present in Alzheimer’s disease (AD). However, the associations between region-specific CC degeneration and homotopic inter-hemispheric FC and their relationships with memory deficits in AD remain uncharacterized. We hypothesized that selective CC degeneration is associated with memory impairment in AD and amnestic mild cognitive impairment (aMCI), which is mediated by homotopic inter-hemispheric functional dysconnectivity. Using structural magnetic resonance imaging (MRI) and task-free functional MRI, we assessed the CC volume and inter-hemispheric FC in 66 healthy controls, 41 aMCI and 41 AD. As expected, AD had CC degeneration and attenuated inter-hemispheric homotopic FC. Nevertheless, aMCI had relatively less severe CC degeneration (mainly in mid-anterior, central, and mid-posterior) and no reduction in inter-hemispheric homotopic FC. The degeneration of each CC sub-region was associated with specific inter-hemispheric homotopic functional disconnections in AD and aMCI. More importantly, impairment of inter-hemispheric homotopic FC partially mediated the association between CC (particularly the central and posterior parts) degeneration and memory deficit. Notably, these results remained after controlling for hippocampal volume. Our findings shed light on how CC degeneration and the related inter-hemispheric FC impact memory impairment in early stage of AD.

Corpus callosum (CC) plays a key role as the primary cortical projection system connecting the two brain hemispheres. CC degeneration, both volume reductions and diffusivity disruptions, has been documented in Alzheimer’s disease (AD)^1^,^2^. Recent evidence suggest an association between CC (and its subregions) degeneration with cognitive impairment in AD^2^. However, the CC changes in amnestic mild cognitive impairment (aMCI), the prodromal stage of AD, remain scarce and controversial. Di Paola and colleagues found reduced white matter density in the anterior CC subregion in aMCI compared to controls^1^. Other studies found splenium of corpus callosum might serve as an early marker to predict progression of aMCI to AD^3^,^4^ (see review^5^). Mixed findings about diffusivity changes of CC in MCI were also reported^1^,^2^,^6^.

CC is the largest commissural white matter bundle in the human brain and the main route for inter-hemispheric transfer of information. Medial temporal lobe damage is associated with memory deficits in AD^7^,^8^. However, emerging evidence suggested that CC might play a secondary role in memory processing mainly due to the need for inter-hemispheric interactions^9^,^10^,^11^,^12^. Previous studies found that participants with agenesis of corpus callosum performed significantly below healthy controls in memory related cognitive tests, suggesting that the corpus callosum facilitates more efficient learning and recall for both verbal and visual information^10^,^12^,^13^. For individuals with CC impairments, visual memory traces in the right hemisphere might be less accessible to the language dominant left hemisphere for verbal recall^10^. Reduced interaction between visual and verbal systems may also affect the richness of initial encoding for both visual and verbal tasks^10^.

In AD, the associations between CC deficits and memory impairment have also been found. Parra and colleagues examined the CC microstructure changes in familial AD based on diffusion MRI^11^. The genu of corpus callosum was found to be responsible for the short-term memory binding impairments while the hippocampal part of cingulum bundle accounted for long-term memory binding deficits in familial AD^11^. Similarly, another study detected a negative correlation between memory and mean diffusivity in the frontal, temporal, or primary sensory subregions of the CC in AD and aMCI^2^. Moreover, based on the network-based neurodegeneration hypothesis^14^, accumulating evidence has shown that the default mode network (DMN) structure and function is damaged in AD^5^,^15^ and the DMN disruptions were associated with memory deficits^16^,^17^,^18^. For example, Bernard and colleagues have demonstrated that dysconnectivity in precuneus, one core region of the DMN (connected by posterior CC)^19^, was related to episodic memory deficits in elderly^20^. CC is known to connect regions of the default mode network^21^ and other intrinsic connectivity networks such as executive control network^22^. CC dysfunction may thus contribute to network dysconnectivity by disrupting the inter-hemispheric homotopic information transfer, leading to memory impairment.

Resting-state or task-free fMRI maps intrinsic functional connectivity within and between large-scale neural networks^23^. Task-free fMRI analysis has detected predictable connectivity reductions in AD^15^,^24^,^25^ and prodromal stage of AD^26^. Because cognitive and behavioral functions rely on interactions between hemispheres and remote brain regions^27^, functional connectivity may clarify fundamental aspects of disease pathophysiology. Moreover, the CC, which primarily connects homologous regions of the cortex, is the major conduit for information transfer between the two cerebral hemispheres. CC degeneration may contribute to the disruption of inter-hemispheric homotopic functional connectivity underlying cognitive deficits seen in AD. However, only few neuroimaging studies have examined inter-hemispheric homotopic functional connectivity in AD and MCI patients and the results are inconsistent^28^,^29^. Using a modular analysis method of graph theory, one study found the insula module, the largest homotopic module in the control group, had lost symmetric functional connection properties in the AD group^28^. More recently, a decreased inter-hemispheric functional connectivity mainly in the anterior brain regions has been reported^29^.

The CC topographically exhibits functional specialization with callosal subregions connecting to different cortical lobes^30^. The genu of the CC connects the orbitofrontal and frontal cortices, whereas the body and the splenium connects the temporal, parietal, and occipital regions^31^. Thus, selective CC subregion degeneration may also be associated with specific disruption of inter-hemispheric homotopic functional connectivity, hence contributing to distinct behavioral and cognitive symptoms in AD and aMCI. Given that functionally linked resting-state networks reflect the underlying structural connectivity^32^,^33^, inter-hemispheric white matter pathway (i.e., CC) abnormalities may influence the functional connectivity patterns in the disease progression of AD^4^,^29^. However, the association between selective CC degeneration, disruption of inter-hemispheric homotopic functional connectivity and memory impairment in AD and aMCI patients remains unclear.

To address these gaps, we combined high resolution structural magnetic resonance imaging (MRI), task-free fMRI and detailed neuropsychological measurements to investigate the degeneration of CC and its subregions, specific disruption of inter-hemispheric homotopic functional connectivity and cognitive impairment in a relatively large sample of AD and aMCI patients. We hypothesized that structural degeneration in posterior CC (compared to anterior) is associated with memory impairment in AD and aMCI. Such relationship may be mediated by the homotopic inter-hemispheric functional disconnections. The purpose of present study is to characterize the associations between region-specific CC degeneration and homotopic inter-hemispheric functional connectivity and their relationships with memory deficits during the prodromal stage of AD.

## Results

### Demographics, hippocampal and clinical characteristics

There were no group differences in gender, race, handedness, head motion, or TIV (Table 1). Group differences in age, mean grey matter-based VMHC and average hippocampal volume were observed (Table 1). The majority (81.8%) of the control subjects had medial temporal lobe atrophy (MTA) scores less than or equal to 1. Almost all (40 out of 41) aMCI patients had MTA scores greater than or equal to 1 and 18 of 41 aMCI patients had MTA scores greater than or equal to 2, 37 of 41 AD patients had MTA scores greater than or equal to 2. Based on a cut-off MTA score of 2 or greater^34^, 37 out of 41 AD and 18 out of 41 aMCI patients fulfilled the National Institute on Aging and the Alzheimer’s Association (NIA-AA) recommended criterion for AD dementia^35^ and MCI due to AD^36^ with intermediate evidence of the AD pathophysiological process.

### Degeneration of corpus callosum in AD and selective degeneration of corpus callosum subregions in aMCI

**Figure 1:**
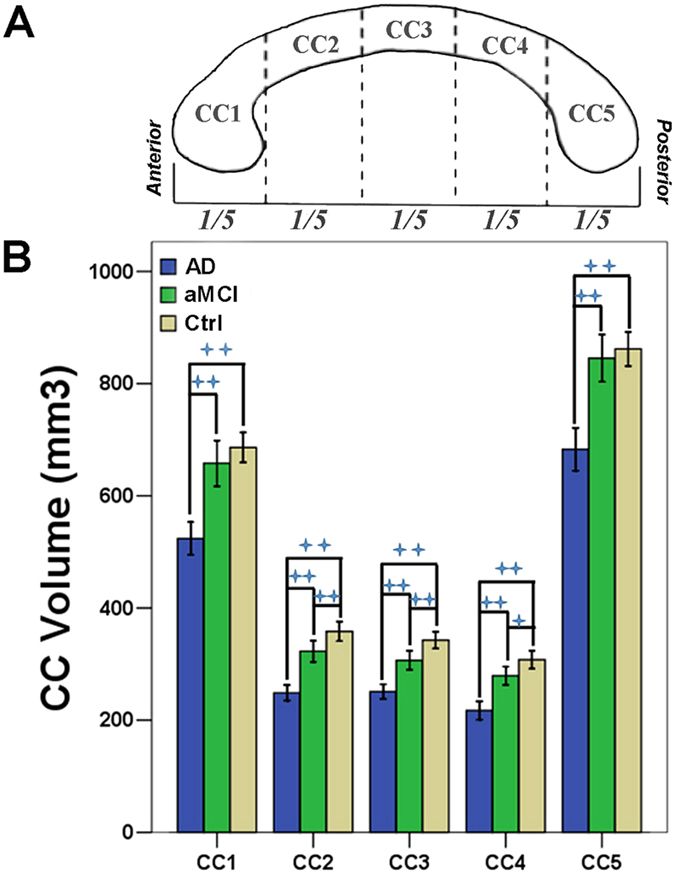
Degeneration of corpus callosum and its sub-regions in AD and amnestic MCI. (**A**) Five sub-regions of corpus callosum was segmented using Freesurfer software. (**B**) After controlling for age, sex, race, handedness and total intracranial volume, AD patients had smaller volume of all five CC sub-regions than aMCI and controls. Patients with aMCI had smaller CC2, CC3 and CC4 sub-regions volume compared to control group. ‘++’ indicates the significance level of p < 0.01 (corrected for multiple comparison) while ‘+’ indicates the significance level of p < 0.05 (corrected for multiple comparison). Abbreviations: AD, Alzheimer’s disease; aMCI, amnestic mild cognitive impairment; Ctrl, Control; CC1, Anterior CC; CC2, mid-anterior CC; CC3, Central CC; CC4, mid-posterior CC; CC5, Posterior CC.

The AD group had smaller volume of the total CC and all its five sub-regions compared to the aMCI and control groups. The aMCI group had smaller total CC, mainly the mid-anterior, central and mid-posterior parts compared to the control group (Fig. 1). In addition, results largely remained when we analyzed the sub-cohort of MCI due to AD-intermediate and AD fulfilling research criterion (Supplementary Table 1).

### Disruption of inter-hemispheric homotopic functional connectivity in AD, but not aMCI, especially in the temporal-parietal regions

**Figure 2:**
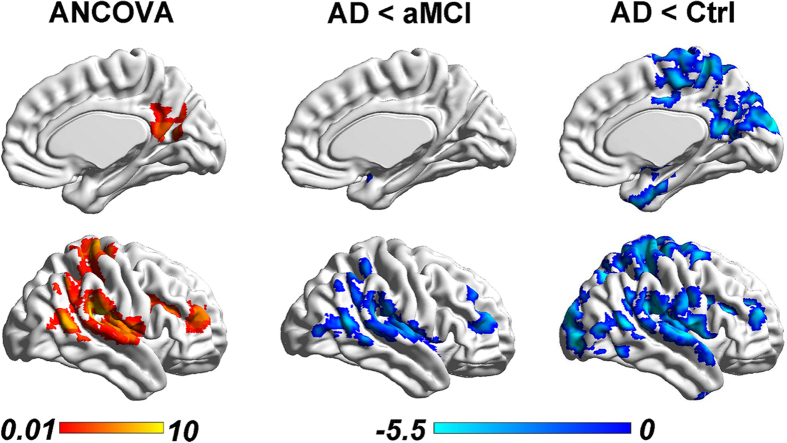
Reduced inter-hemispheric homotopic functional connectivity in AD and aMCI. Left: After controlling for age, sex, race, handedness, and head motion, the three groups showed significant differences in the inter-hemispheric homotopic functional connectivity (i.e., VMHC) in the MFG, PreC, PoCG and ROL (regions highlighted in orange color) using ANOVA (color bar represents F-values). Middle: The AD patients had decreased VMHC in the MFG and temporal-parietal regions (extending to the insula) (regions highlighted in blue color) compared to aMCI patients. Right: The AD patients had decreased VMHC in more widespread brain regions, including the MFG, temporal-parietal and occipital regions (extending to the PCC, insula and hippocampus), than the controls. The blue color bars indicate t-values of each comparison. No differences in VMHC were detected between aMCI and controls. All results were reported at a height threshold of p < 0.01 and cluster threshold of p < 0.05 with GRF correction. Abbreviations: AD, Alzheimer’s disease; aMCI, amnestic mild cognitive impairment; Ctrl, Control; MFG, Middle frontal gyrus; ROL, Rolandic; PoCG, Postcentral gyrus; PreC, Precuneus; PCC, Posterior cingulate cortex; VMHC, voxel mirrored homotopic connectivity; GRF, Gaussian random field.

AD patients had lower inter-hemispheric homotopic functional connectivity between the bilateral middle frontal gyrus (MFG), middle temporal gyrus (MTG), superior temporal gyrus (STG) extending to bilateral hippocampus and posterior cingulate cortex regions, middle occipital gyrus (MOG), and bilateral postcentral gyrus (PoCG), precentral gyrus (PreCG) compared with the controls (Fig. 2, right panel and Supplementary Table 2). Compared to aMCI patients, AD patients had a lower inter-hemispheric homotopic functional connectivity in similar regions, including bilateral MFG, STG, extending to the bilateral inferior parietal lobe (IPL) (Fig. 2, middle panel and Supplementary Table 2). No differences between aMCI and controls were found (Supplementary Fig. 1 and Table 2. No increase in inter-hemispheric homotopic functional connectivity was found in AD patients. Moreover, the observed group differences in inter-hemispheric homotopic functional connectivity remained the same when we repeated the analyses in an age-matched and right-handers only sub-cohort (Supplementary Results, Supplementary Fig. 2 and Table 3) and in a sub-cohort with ‘MCI due to AD-Intermediate likelihood’ and AD fulfilling research criterion (Supplementary Fig. 3). We also repeated the analysis by including subjects with memory impairment on neuropsychological assessments but without subjective memory complaints, i.e., amnestic cognitive impairment no dementia (a-CIND), the results were similar to the primary findings (Supplementary Results, Supplementary Fig. 4 and Table 4).

### CC degeneration correlates with inter-hemispheric homotopic functional connectivity in AD and aMCI

**Figure 3:**
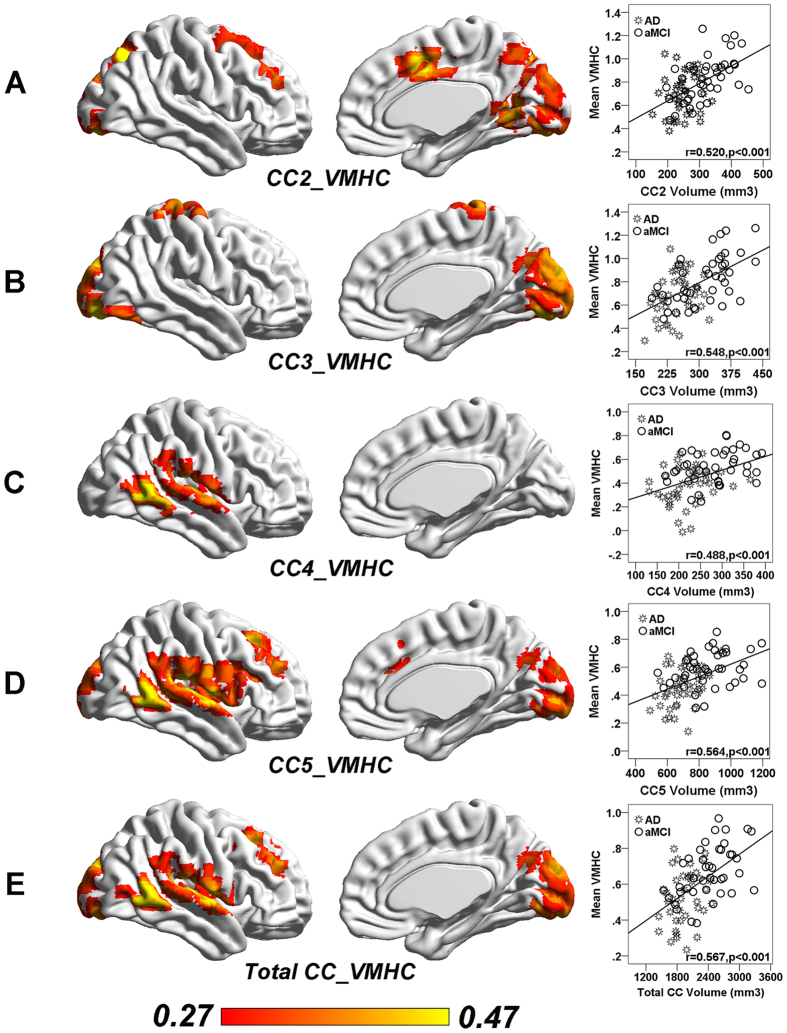
Reduced corpus callosum volume is associated with disrupted inter-hemispheric homotopic functional connectivity in AD and aMCI patients. (**A**) CC2 sub-region volume showed a significantly positive correlation with the VMHC in IOG and SMA in patients with aMCI and AD. (**B**) CC3 sub-region volume showed a significantly positive correlation with the VMHC in PoCG and CUN in aMCI and AD. (**C**) CC4 sub-region volume showed a significantly positive correlation with the VMHC in MTG in aMCI and AD. (**D**) CC5 sub-region volume showed a significantly positive correlation with the VMHC in CAL, MTG and MFG in aMCI and AD. (**E**) CC total volume showed a significantly positive correlation with the VMHC in MTG, MFG and CUN in aMCI and AD. All results were reported at a height threshold of p < 0.01 and cluster threshold of p < 0.05 with GRF correction. (GRF corrected, individual p < 0.01, cluster p < 0.05). The right column presents the scatterplots of CC volume and corresponding mean VMHC across all patients with aMCI and AD (p < 0.001 after multiple comparison correction). CC2, mid-anterior CC; CC3, Central CC; CC4, mid-posterior CC; CC5, Posterior CC. Abbreviations: Ctrl, Control; AD, Alzheimer’s disease; aMCI, amnestic mild cognitive impairment; AAL, Anatomical Automatic Labeling; MNI, Montréal Neurological Institute; MCC, Middle cingulate cortex; MTG, Middle temporal gyurs; SOG, Superior occipital gyrus; SMA, Supplement motor area; IOG, Inferior occipital gyrus; SPG, Superior parietal gyrus; CUN, Cuneus; CAL, Calcarine; STG, superior temporal gyrus.

The total CC volume showed positive correlation with the frontal (SFG and MFG), temporal-parietal (MTG, STG, ROL and SMG), and occipital (CUN, LING, CAL and LOG) VMHC in AD and aMCI patients (Fig. 3 and Supplementary Table 5). Anterior CC volume had no association with inter-hemispheric homotopic functional connectivity. Mid-anterior and central CC volume was associated with VMHC in the frontal (SMA, PoCG) and occipital (IOG) regions while mid-posterior and posterior CC volumes are associated with VMHC in the temporal (MTG, STG), frontal (MFG) and occipital (CAL) regions (Fig. 3 and Supplementary Table 5). In addition, these associations largely remained when we analyzed the sub-cohort of MCI due to AD-intermediate and AD fulfilling research criterion (a joint height and cluster threshold of p < 0.05 with GRF correction) (Supplementary Table 6).

### The impact of CC degeneration on memory deficits is mediated by inter-hemispheric functional connectivity

The volume of mid-anterior, central and posterior sub-regions and total CC were associated with memory in AD and aMCI (p < 0.05 corrected for multiple comparisons). These associations still remained when we controlled for hippocampal volume (Supplementary Table 7). No associations with memory were found in anterior CC volume.

**Figure 4:**
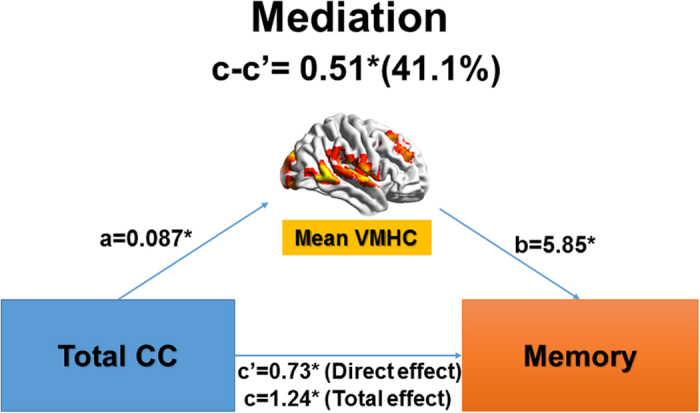
Inter-hemispheric homotopic functional connectivity mediated the association between corpus callosum degeneration and memory impairment in AD and aMCI. Regions whose inter-hemispheric homotopic functional connectivity (i.e., VMHC) was related to total corpus callosum (CC) volume (Fig. 3E) mediated the impact of total CC degeneration on memory deficit in AD and aMCI. ‘c’ denotes the total effect of CC volume on memory; ‘c” denotes the direct effect of CC volume on memory (not through inter-hemispheric homotopic functional connectivity); and ‘c-c” denotes the indirect effect (mediated effect, through inter-hemispheric homotopic functional connectivity). ‘*’ Means the significance level of p < 0.05.

One step further, we found inter-hemispheric functional connectivity partially (41.1%) mediated the association between total CC and memory in AD and aMCI patients (Fig. 4). Such mediation effects were largely similar after controlling for hippocampal volume (Supplementary Fig. 5). Inter-hemispheric functional connectivity also partially mediated the associations of each CC sub-region (central and posterior) with memory in AD and aMCI patients (Supplementary Fig. 6).

## Discussion

Our findings confirmed that CC degeneration and attenuated inter-hemispheric homotopic functional connectivity was present in AD. However, aMCI patients had less severe CC degeneration (particularly in the mid-anterior, central and mid-posterior parts) and no reductions in inter-hemispheric homotopic functional connectivity. The degeneration of each CC sub-region was associated with specific inter-hemispheric homotopic functional disconnections. More importantly, impairment of inter-hemispheric homotopic functional connectivity partially mediated the association between CC degeneration and memory deficits in patients with AD or aMCI. Notably, these results remained after controlling for hippocampal volume. These findings provide novel insight into the impact of CC and its related inter-hemispheric functional connectivity on memory impairment in early AD.

Previous studies suggested that AD and MCI patients had a lower midsagittal area or thickness and volume of CC when compared with controls^1^,^37^,^38^. However, evidence on degeneration of specific CC subregions in MCI was inconsistent. One group reported smaller anterior part of the CC (rostrum and genu) in patients with MCI^38^, while another study showed a reduction in the posterior region (isthmus and splenium) relative to healthy controls^37^. These inconsistencies may be due to the heterogeneity of the sample; for example, some but not all MCI patients in previous studies were of the amnestic subtype. To minimize such concern, we have carefully evaluated all patients and found selective mid-anterior, central, and mid-posterior atrophy of CC in amnestic MCI patients only. Moreover, previous CC studies in MCI typically involved area measurements of the most medial sagittal slice of the MRI. However, measures of the callosal area are likely to reflect axon density in the genu and anterior splenium but not the axon density in the posterior midbody and splenium^39^. We adopted the freesurfer approach of CC volume segmentation to minimize the possible bias when comparing different CC subregions. Our findings of selective CC degeneration in aMCI correspond well with the typical selective grey matter atrophy pattern in AD. AD features atrophy mainly in a network including posterior parietal, hippocampus, and medial temporal regions^14^. The inter-hemispheric connectivity between these regions is mainly through the central and mid-posterior parts of the CC^2^. We speculate that the white matter degeneration of central and mid-posterior CC might partly contribute to or even accelerate the progression of grey matter atrophy during the progression from aMCI to AD^40^. Furthermore, CC is the key white matter pathways linking cortical and subcortical hubs of the left and right hemispheres together. These hubs are preferentially vulnerable to Aβ deposition^41^, atrophy^42^, and disruption of activity^24^. A recent study found that the anatomical distance via white matter pathways to ourbreak regions modulates the Aβ propagation processes^43^. Therefore, CC degeneration is likely related to the neuropathological injury and network-based breakdown in AD and aMCI.

As expected, AD patients had reductions in inter-hemispheric homotopic functional connectivity, especially in the temporal-parietal regions. This is consistent with previous evidence of temporal-parietal abnormalities in AD based on pathology^44^ and multimodal imaging models^45^,^46^,^47^. In addition, AD had reduced inter-hemispheric homotopic functional connectivity in the PoCG, PreCG, and MFG, which may relate to the gross and fine motor skill impairments^48^,^49^ and working memory deficits^50^. Amyloid-β plaques and phosphorylated tau tangles are the hallmark neuropathology markers of AD. Cerebral spinal fluid (CSF) markers of amyloid deposition and neuronal injury in early AD are associated with a dual pattern of cortical network disruption, affect key regions of the default mode network and the temporal cortex^51^. We found inter-hemispheric disconnection between bilateral frontal, temporal and parietal regions, which were particular involved in amyloid deposition and tau protein-related neuronal injury^52^. It is possible that the inter-hemispheric functional disconnection observed here may be associated with or even accelerate the network-based neurodegeneration^14^,^40^, particularly between homotopic regions within one network or from initial disease epicenters to the rest of the target network.

Interestingly, patients with aMCI had no changes in inter-hemispheric homotopic functional connectivity compared to healthy controls. This finding suggests that the inter-hemispheric homotopic functional connectivity is maintained in the pre-dementia stage. However, given evidence of CC subregional degeneration in aMCI, it is plausible that CC degeneration may precede the disruption of inter-hemispheric homotopic functional connectivity in the disease progression of AD. Despite of selective CC degeneration, patients at early stage of the disease might still be able to maintain inter-hemispheric homotopic functional connectivity to support major cognitive functions. Additional brain regions might be recruited to create new functional connections for task performance^53^,^54^. These compensatory efforts can be accompanied by re-organization of intrinsic connectivity networks to achieve optimal efficiency^55^,^56^. With further progression in disease, these mechanisms appear to eventually fail^57^, as evidenced by reduced inter-hemispheric homotopic functional connectivity, accompanied with more severe CC damage.

Each CC sub-region degeneration was associated with specific patterns of inter-hemispheric homotopic functional connectivity disruptions in AD and aMCI patients. Previous studies suggested robust links between structural and functional connectivity in the human brain^33^,^58^,^59^. As expected, the mid-anterior CC subregions correlated with the inter-hemispheric homotopic functional connectivity of bilateral frontal cortex, central CC subregion correlated with the inter-hemispheric connectivity of bilateral PoCG, and the mid-posterior and posterior CC subregions correlated with the interhemispheric homotopic functional connectivity of the temporal and occipital cortices. Indeed, specific CC fibers topographically correlate with specific cortical connectivity^2^,^60^.

Selective CC subregion degeneration and specific disruption of inter-hemispheric functional connectivity was correlated with poorer performance in memory in AD and aMCI patients. Memory impairment is associated with neuropathology within the hippocampal/medial temporal lobe^8^. In addition, based on present results and recent literature^9^,^10^, inter-hemispheric interactions might play a secondary role in memory impairment. More importantly, we showed that inter-hemispheric homotopic functional connectivity mediated up to 41.1% of the associations of CC degeneration and memory in AD and aMCI. Notably, the mediation effect preserved even after controlling for hippocampal volume. To our knowledge, this is the first study demonstrating such mediation effect. CC degeneration alone may not result in severe memory impairment, as functional connectivity might still be able to compensate. Disruption of inter-hemispheric homotopic functional connectivity accompanied with CC degeneration would largely disrupt large-scale network interactions (both functional specialization and segregation) and therefore lead to memory impairment in AD and aMCI^61^. Furthermore, our findings suggest that the degeneration of CC might influence cognition through other mechanisms, because inter-hemispheric homotopic functional connectivity only partially mediated such effect. Other abnormalities in intra-hemispheric or inter-hemispheric heterotopic functional connectivity (interhemispheric connections between heterotopic regions) and network architecture might be associated with CC degeneration, which in turn exerts additional impact on cognition^62^,^63^. Taken together, these findings highlight the importance of inter-hemispheric coherence; deteriorations of such structural and functional inter-hemispheric commutations would largely impact memory functions. Further developed, CC degeneration and the related inter-hemispheric functional connectivity abnormalities might be helpful for early identification and progression prediction in AD. However, though recent study demonstrated brain activity (connectivity) in the “resting” state when subjects were not performing any explicit task can predict differences in fMRI activation across a range of cognitive paradigms^64^, the extrinsic and intrinsic functional architectures of the human brain are not equivalent^65^. Future studies combining resting-state and task-based fMRI would shed more lights on brain-cognition associations.

Several limitations should be considered. First, a standard symmetrical brain template was used for the calculation of voxel-based homotopic inter-hemispheric functional connectivity. We applied spatial smoothing to improve the spatial correspondence between homotopic areas and minimize the potential confounder of inter-subject anatomical variability. Second, FreeSurfer method to equally sub-divide the CC might not reflect the true fiber compositions in each subregion accurately. Third, the patient and control groups differed in age. However, we included age as a variable in all analyses to account for the effect of age. Moreover, similar results were found in a subset of participants that were age-matched across the three groups. Given that the relatively low temporal resolution of the BOLD-fMRI might prevent us from detecting/observing fast cognitive processes accurately, multimodal neuroimaging studies such as concurrent EEG-fMRI might provide complementary insights on brain-cognition relationship and its vulnerability patterns in disease^66^. Lastly, our mediation and correlation analyses were based on cross-sectional data. Future longitudinal or lesion studies are needed to investigate the temporal dynamics or the possible causal relationships between CC degeneration, inter-hemispheric functional disconnections, and cognitive performance in disease progression^67^.

## Conclusion

In sum, to our knowledge, this is the first study to demonstrate associations between region-specific CC degeneration and homotopic inter-hemispheric functional dysconnectivity, as well as their relationships with memory deficits in AD and aMCI. All CC subregions had degeneration in AD while only mid-anterior, central and mid-posterior parts exhibited shrinkage in aMCI. Homotopic inter-hemispheric functional connectivity disruption was detected in AD but not aMCI, suggesting possible compensatory mechanism. Intriguingly, impairment of inter-hemispheric homotopic functional connectivity partially mediated the associations between CC degeneration and memory deficits. These findings provide novel insights on the impact of brain inter-hemispheric structural and functional changes on memory impairment in early stage of AD.

## Methods

### Participants

Ethics approval was obtained from the SingHealth Institutional Review Board and the National Healthcare Group Domain-Specific Review Board. The study was conducted in accordance with the Declaration of Helsinki. Written informed consent was obtained prior to recruitment. Patients with AD and aMCI recruited from two study sites in Singapore (i.e., memory clinics from National University Hospital and Saint Luke’s Hospital). Controls were recruited from both the memory clinics and the nearby communities. Details on participants’ recruitment and selection are in the Supplementary Methods. Briefly, out of 411 recruited participants, we included 66 healthy controls, 41 aMCI, and 41 AD patients in the present study.

### Neuropsychological measurements and diagnosis

Trained research psychologists administered brief cognitive screening tests, the Mini-Mental State Examination (MMSE), the Montreal Cognitive Assessment (MoCA), the informant questionnaire on cognitive decline and a formal neuropsychological battery locally validated for older Singaporeans^68^,^69^ (Supplementary Methods). Diagnoses of cognitive impairment and dementia were made at weekly consensus meetings attended by study clinicians and neuropsychologists. Clinical features, blood investigations, psychometrics and neuroimaging data were reviewed. Diagnostic definitions of amnestic MCI and AD and derivation of domain special cognitive function Z-scores can be found in our previous work^69^ (see details in Supplementary Methods)^34^.

### Imaging acquisition

All images were collected using a 3T Siemens Tim Trio system (Siemens, Erlangen, Germany). Each participant underwent a T1-weighted structural MRI and a task-free fMRI in the same session. Imaging parameters are presented in Supplementary Methods.

### Visual rating of medial temporal lobe atrophy

Following our previous work^70^, the medial temporal lobe atrophy (MTA) was rated on the coronal T1-weighted images in their native space by a 5-point visual rating scale based on the height of the hippocampal formation, the width of the temporal horn, and the width of the choroids fissure^34^. Grade 0 indicates no MTA and Grade 4 indicates the most severe atrophy. All images were rated by a neurologist who was blind to the subjects’ clinical information. MTA scores of the left and right hippocampi were averaged to present the MTA grade of a subject^71^. We used MTA greater or equal to 2 as the cut-off for hippocampal atrophy^34^ in present study.

### The corpus callosum and hippocampal volume calculation

We calculated the volume of the corpus callosum (CC) and its subregions, and hippocampal volume using the FreeSurfer software package (http://surfer.nmr.mgh.harvard.edu/, v.5.3) following previous approach^72^. The automated procedures for subcortical volume measurements of different brain structures have been described previously^73^ (see details in Supplementary Methods). This FreeSurfer-based automatic procedure is comparable with manual segmentation^73^. The volume of each brain structure was then calculated by summing up all voxels within the structure.

The CC was automatically identified and segmented into 5 segments using the FreeSurfer processing software following previous work^72^, with each section representing a fifth of the total area/volume that broadly corresponds to functional subdivisions^74^, including anterior (CC1), mid-anterior (CC2), central (CC3), mid-posterior (CC4) and posterior portions (CC5; Fig. 1A). The total CC volume was calculated as the sum of the 5 segments.

Anatomic volume of the bilateral hippocampus was delineated using information on image intensity, probabilistic atlas location and spatial relationships between subcortical structures^73^,^75^. Following our previous work^76^, visual inspection and edition were performed, where necessary, using standard procedures (http://surfer.nmr.mgh.harvard.edu/fswiki/Edits), blind to diagnostic status. We evaluated the between-group differences in the average hippocampal volume (left and right) using ANCOVA, with age and total intracranial volume as covariates.

### Task-free fMRI data preprocessing

Task-free fMRI data preprocessing was performed using Data Processing & Analysis of Brain Imaging (DPABI 1.2)^77^ based on statistical parametric mapping (SPM8, http://www.fil.ion.ucl.ac.uk/spm), following our previous approaches^67^,^78^,^79^ (Supplementary Methods for details).

After quality control, all participants had the minimum absolute translation and rotation (not exceeding 1.5 mm or 1.5°). Moreover, the mean framewise displacement (FD) was computed by averaging the FD from every time point for each subject and included as a covariate in statistical analyses^80^.

### Voxel-mirrored homotopic functional connectivity derivation

We computed the subject-level VMHC following previous approach detailed in Zuo *et al.*^81^ using DPABI software. For each subject, the homotopic functional connectivity was computed as the Pearson’s correlation coefficient between each voxel’s residual time series and that of its symmetrical inter-hemispheric counterpart. Correlation values were then Fisher’s z-transformed to produce subject-specific VMHC z-score maps for further statistical analyses.

### Statistical analysis

#### Group differences

One-way ANOVA analyses on six volume indices of CC (total CC and 5 sub-regions), demographics, neuropsychological data and motion parameters were performed to determine group differences. χ^2^-test was used for group comparisons of gender, handedness and race. The results were reported at the significance level of p < 0.05 (two-tailed) and corrected for multiple comparisons. All statistical analyses were performed using SPSS software (v.23.0, IBM, USA).

Group differences in VMHC were determined by performing whole-brain voxelwise ANCOVA analysis on subject-specific VMHC maps, with age, handedness, gender, ethnicity and head motion as nuisance variables. The results were reported at a height threshold of p < 0.01 and cluster threshold of p < 0.05, with Gaussian random field (GRF) correction^82^, with a gray matter mask produced by the group-level symmetrical template average across all subjects. We repeated all the group comparisons of CC and VMHC in a sub-cohort of participants fulfilling research criterion.

#### Associations between CC volume and VMHC

To evaluate the relationship between structural inter-hemispheric integrity (CC volume) and functional inter-hemispheric connectivity (VMHC), we performed a whole-brain voxel-based correlation analysis of VMHC against six CC volume indices separately across all patients (AD and aMCI). Age, handedness, gender, ethnicity and total intracranial volume (TIV) were entered as covariates. Positive and negative correlations were reported at a single voxel height of p < 0.01 and a cluster p < 0.05 with GRF correction. We repeated the association analysis in the sub-cohort of MCI due to AD-intermediate and AD fulfilling research criterion.

#### Associations between inter-hemispheric connectivity and memory

To test the associations between brain inter-hemispheric coherence and memory deficits in AD and aMCI patients, we performed correlation analyses between brain measures (VMHC and CC volume) and memory scores in SPSS. First, for each subject, the mean VMHC values of regions showing group differences in ANCOVA analyses were extracted separately using REST^83^ (http://resting-fmri.sourceforge.net) (by averaging the VMHC values of all voxels within each region). Pearson’s correlations were then computed between the six CC volume indices, mean VMHC values of each region, and the memory scores. The results were reported at a significance level of p < 0.05 with Bonferroni correction for multiple comparisons (6 CC volume indices; 4 VMHC regions).

#### Mediation analysis of VMHC, CC volume, and memory

To investigate whether the influence of CC degeneration on memory dysfunction, the dominant symptom in AD and aMCI, was mediated by inter-hemispheric homotopic functional connectivity (VMHC values) in AD and aMCI patients, we performed a mediation analysis with each CC volume as the independent variable, memory domain z-scores as dependent variable, and mean VMHC of those regions correlated with CC volume as the mediato^84^ using SPSS software. The mean VMHC values were calculated by averaging the VMHC values of all the voxels showing positive correlations with each CC volume in the whole-brain voxel-wise VMHC-CC association analyses. We repeated the association analysis (inter-hemispheric connectivity and memory) and the mediation analysis with hippocampal volume as an additional covariate.

## Additional Information

**How to cite this article**: Qiu, Y. *et al.* Inter-hemispheric functional dysconnectivity mediates the association of corpus callosum degeneration with memory impairment in AD and amnestic MCI. *Sci. Rep.* **6**, 32573; doi: 10.1038/srep32573 (2016).

## Supplementary Material

Supplementary Information

## Figures and Tables

**Table 1:** Demographic, neuropsychological and neuroimaging characteristics.

	Ctrl (n = 66)	aMCI (n = 41)	AD (n = 41)	P
Age	67.2 (5.98)	72.1 (7.67)^c^	75.8 (8.14)^mc^	<0.001**
Gender (M/F)	27/39*	19/22*	16/25*	0.780
Race (Chinese/Malay)	62/4*	34/7*	37/4*	0.185
Handiness (R/L)	60/6*	38/3*	41/0*	0.149
CDRSum^a^	0.1 (0.22)	0.85 (0.63)^c^	6.2 (2.28)^mc^	<0.0001**
CDRGlobal^a^	0.1 (0.20)	0.4 (0.20)^c^	1.1 (0.33)^mc^	<0.0001**
MMSE^a^	27.4 (1.94)	24.4 (3.62)^c^	16.4 (5.07)^mc^	<0.0001**
MoCA^a^	25.2 (2.88)	19.5 (4.73)^c^	14.3 (5.54)^mc^	<0.0001**
Memory^a^	10.84 (1.96)	6.17 (2.35)^c^	2.61 (1.95)^mc^	<0.0001**
EDFABScore^a^	16.1 (1.47)	14.0 (2.64)^c^	9.9 (3.74)^mc^	<0.0001**
AttentionStatus^a^	8.36 (1.006)	6.89 (1.43)^c^	5.01 (2.506)^mc^	<0.0001**
LangStatus^a^	16.12 (2.300)	13.23 (2.317)^c^	8.56 (3.182)^mc^	<0.0001**
VisConStatus^a^	20.51 (3.159)	16.33 (4.038)^c^	10.19 (5.697)^mc^	<0.0001**
VisMotorStatus^a^	35.47 (14.582)	39.95 (23.578)	54.63 (28.545)^mc^	0.003**
Head motion^b^	0.109 (0.077)	0.093 (0.0530)	0.107 (0.0581)	0.580
VMHC^a^	0.581 (0.1205)	0.557 (0.1222)	0.525 (0.1155)^mc^	0.049**
TIV (mm^3^)^a^	1.5E + 6 (1.49E + 5)	1.4E + 6 (1.81E + 5)	1.4E + 6 (1.47E + 5)	0.582
Hippocampal volume (mm^3^)^d^	3800.8 (62.74)	3279.7 (75.30)^c^	2806.5 (80.96)^mc^	<0.0001**

Unless otherwise indicated, values represent the mean ± standard deviation. Measures marked with ‘*’ indicate that χ^2^ test was used. Superscript letters indicate whether group mean was significantly worse than healthy control (Ctrl) (c), amnestic mild cognitive impairment (aMCI) (m) based on post hoc pairwise comparisons (p < 0.05). The last column represents the p-values of Analysis of covariance (ANCOVA) between groups; ‘**’ indicates significant differences between the three groups at the threshold of p < 0.05. Abbreviations: Ctrl, Control; aMCI, amnestic mild cognitive impairment; AD, Alzheimer’s disease; MMSE, Mini Mental State Examination; CDR, Clinical Dementia Rating scale.

^a^We adjusted for age when performing the group comparisons.

^b^Head motion was indexed by mean framewise displacement (FD) derived with Jenkinson’s relative root mean square (RMS) algorithm^85^.

^d^Average volume of left and right hippocampus. We adjusted for age and TIV when performing the group comparisons.

## References

[b1] Di Paola M. *et al.* When, where, and how the corpus callosum changes in MCI and AD: a multimodal MRI study. Neurology 74, 1136–1142, doi: 10.1212/WNL.0b013e3181d7d8cb (2010). 20368633

[b2] Wang P. N. *et al.* Callosal degeneration topographically correlated with cognitive function in amnestic mild cognitive impairment and Alzheimer’s disease dementia. Hum Brain Mapp 35, 1529–1543, doi: 10.1002/hbm.22271 (2014). 23670960 PMC6869324

[b3] Carmeli C. *et al.* Demyelination in mild cognitive impairment suggests progression path to Alzheimer’s disease. PloS one 8, e72759 (2013). 24023644 10.1371/journal.pone.0072759 PMC3758332

[b4] Filippi M. & Agosta F. Structural and functional network connectivity breakdown in Alzheimer’s disease studied with magnetic resonance imaging techniques. Journal of Alzheimer’s disease : JAD 24, 455–474, doi: 10.3233/JAD-2011-101854 (2011). 21297259

[b5] Teipel S. *et al.* Measuring Cortical Connectivity in Alzheimer’s Disease as a Brain Neural Network Pathology: Toward Clinical Applications. J Int Neuropsychol Soc 22, 138–163, doi: 10.1017/s1355617715000995 (2016). 26888613

[b6] Stahl R. *et al.* White Matter Damage in Alzheimer Disease and Mild Cognitive Impairment: Assessment with Diffusion-Tensor MR Imaging and Parallel Imaging Techniques. Radiology 243, 483–492, doi: 10.1148/radiol.2432051714 (2007). 17456872

[b7] Hyman B. T., Damasio A. R., Van Hoesen G. W. & Barnes C. L. Alzheimer’s disease: cell-specific pathology isolates the hippocampal formation. Science 298, 83–95 (1984). 10.1126/science.6474172 6474172

[b8] Brown T. I., Staresina B. P. & Wagner A. D. Noninvasive Functional and Anatomical Imaging of the Human Medial Temporal Lobe. Cold Spring Harbor perspectives in biology 7, a021840 (2015). 25780085 10.1101/cshperspect.a021840 PMC4382742

[b9] Christman S. D., Propper R. E. & Dion A. Increased interhemispheric interaction is associated with decreased false memories in a verbal converging semantic associates paradigm. Brain and cognition 56, 313–319 (2004). 15522769 10.1016/j.bandc.2004.08.005

[b10] Erickson R. L., Paul L. K. & Brown W. S. Verbal learning and memory in agenesis of the corpus callosum. Neuropsychologia 60, 121–130, doi: 10.1016/j.neuropsychologia.2014.06.003 (2014). 24933663 PMC4337878

[b11] Parra M. A. *et al.* Memory binding and white matter integrity in familial Alzheimer’s disease. Brain 138, 1355–1369, doi: 10.1093/brain/awv048 (2015). 25762465 PMC5963407

[b12] Paul L. K., Erickson R. L., Hartman J. A. & Brown W. S. Learning and memory in individuals with agenesis of the corpus callosum. Neuropsychologia 86, 183–192, doi: 10.1016/j.neuropsychologia.2016.04.013 (2016). 27091586

[b13] Siffredi V., Anderson V., Leventer R. J. & Spencer-Smith M. M. Neuropsychological profile of agenesis of the corpus callosum: a systematic review. Dev Neuropsychol 38, 36–57, doi: 10.1080/87565641.2012.721421 (2013). 23311314

[b14] Seeley W. W., Crawford R. K., Zhou J., Miller B. L. & Greicius M. D. Neurodegenerative diseases target large-scale human brain networks. Neuron 62, 42–52 (2009). 19376066 10.1016/j.neuron.2009.03.024 PMC2691647

[b15] Zhou J. *et al.* Divergent network connectivity changes in behavioural variant frontotemporal dementia and Alzheimer’s disease. Brain 133, 1352–1367 (2010). 20410145 10.1093/brain/awq075 PMC2912696

[b16] Koshino H., Minamoto T., Yaoi K., Osaka M. & Osaka N. Coactivation of the Default Mode Network regions and Working Memory Network regions during task preparation. Sci Rep 4, 5954, doi: 10.1038/srep05954 (2014). 25092432 PMC4121601

[b17] Ward A. M. *et al.* The parahippocampal gyrus links the default-mode cortical network with the medial temporal lobe memory system. Hum Brain Mapp 35, 1061–1073, doi: 10.1002/hbm.22234 (2014). 23404748 PMC3773261

[b18] Andrews-Hanna J. R. *et al.* Disruption of large-scale brain systems in advanced aging. Neuron 56, 924–935, doi: 10.1016/j.neuron.2007.10.038 (2007). 18054866 PMC2709284

[b19] Cavanna A. E. & Trimble M. R. The precuneus: a review of its functional anatomy and behavioural correlates. Brain 129, 564–583, doi: 10.1093/brain/awl004 (2006). 16399806

[b20] Bernard C. *et al.* PCC characteristics at rest in 10-year memory decliners. Neurobiology of Aging, doi: 10.1016/j.neurobiolaging.2015.07.002 (2015). 26234756

[b21] Teipel S. J. *et al.* White matter microstructure underlying default mode network connectivity in the human brain. Neuroimage 49, 2021–2032 (2010). 19878723 10.1016/j.neuroimage.2009.10.067

[b22] van den Heuvel M. P., Mandl R. C., Kahn R. S., Pol H. & Hilleke E. Functionally linked resting‐state networks reflect the underlying structural connectivity architecture of the human brain. Human brain mapping 30, 3127–3141 (2009). 19235882 10.1002/hbm.20737 PMC6870902

[b23] Fox M. D. & Raichle M. E. Spontaneous fluctuations in brain activity observed with functional magnetic resonance imaging. Nat Rev Neurosci 8, 700–711 (2007). 17704812 10.1038/nrn2201

[b24] Greicius M. D., Srivastava G., Reiss A. L. & Menon V. Default-mode network activity distinguishes Alzheimer’s disease from healthy aging: evidence from functional MRI. Proc Natl Acad Sci USA 101, 4637–4642, doi: 10.1073/pnas.0308627101 (2004). 15070770 PMC384799

[b25] Supekar K., Menon V., Rubin D., Musen M. & Greicius M. D. Network analysis of intrinsic functional brain connectivity in Alzheimer’s disease. PLoS Comput Biol 4, e1000100 (2008). 18584043 10.1371/journal.pcbi.1000100 PMC2435273

[b26] Sorg C. *et al.* Selective changes of resting-state networks in individuals at risk for Alzheimer’s disease. Proceedings of the National Academy of Sciences 104, 18760–18765, doi: 10.1073/pnas.0708803104 (2007). PMC2141850 18003904

[b27] Mesulam M. M. From sensation to cognition. Brain 121 (Pt 6), 1013–1052 (1998). 9648540 10.1093/brain/121.6.1013

[b28] Chen G. *et al.* Modular reorganization of brain resting state networks and its independent validation in Alzheimer’s disease patients. Front Hum Neurosci 7, 456, doi: 10.3389/fnhum.2013.00456 (2013). 23950743 PMC3739061

[b29] Wang Z. *et al.* Interhemispheric Functional and Structural Disconnection in Alzheimer’s Disease: A Combined Resting-State fMRI and DTI Study. PLoS One 10, e0126310, doi: 10.1371/journal.pone.0126310 (2015). 25938561 PMC4418835

[b30] De Lacoste M. C., Kirkpatrick J. B. & Ross E. D. Topography of the human corpus callosum. J Neuropathol Exp Neurol 44, 578–591 (1985). 4056827 10.1097/00005072-198511000-00004

[b31] Abe O. *et al.* Topography of the Human Corpus Callosum Using Diffusion Tensor Tractography. Journal of Computer Assisted Tomography 28, 533–539 (2004). 15232387 10.1097/00004728-200407000-00016

[b32] Honey C. *et al.* Predicting human resting-state functional connectivity from structural connectivity. Proceedings of the National Academy of Sciences 106, 2035–2040 (2009). 10.1073/pnas.0811168106 PMC2634800 19188601

[b33] Greicius M. D., Supekar K., Menon V. & Dougherty R. F. Resting-state functional connectivity reflects structural connectivity in the default mode network. Cerebral cortex 19, 72–78, doi: 10.1093/cercor/bhn059 (2009). 18403396 PMC2605172

[b34] Scheltens P. *et al.* Atrophy of medial temporal lobes on MRI in “probable” Alzheimer’s disease and normal ageing: diagnostic value and neuropsychological correlates. Journal of Neurology, Neurosurgery, and Psychiatry 55, 967–972 (1992). 10.1136/jnnp.55.10.967 PMC1015202 1431963

[b35] McKhann G. M. *et al.* The diagnosis of dementia due to Alzheimer’s disease: Recommendations from the National Institute on Aging-Alzheimer’s Association workgroups on diagnostic guidelines for Alzheimer’s disease. Alzheimer’s & Dementia 7, 263–269 (2011). 10.1016/j.jalz.2011.03.005 PMC3312024 21514250

[b36] Albert M. S. *et al.* The diagnosis of mild cognitive impairment due to Alzheimer’s disease: Recommendations from the National Institute on Aging-Alzheimer’s Association workgroups on diagnostic guidelines for Alzheimer’s disease. Alzheimer’s & dementia 7, 270–279 (2011). 10.1016/j.jalz.2011.03.008 PMC3312027 21514249

[b37] Wang P. J. *et al.* Regionally specific atrophy of the corpus callosum in AD, MCI and cognitive complaints. Neurobiol Aging 27, 1613–1617, doi: 10.1016/j.neurobiolaging.2005.09.035 (2006). 16271806 PMC3482483

[b38] Thomann P. A., Wustenberg T., Pantel J., Essig M. & Schroder J. Structural changes of the corpus callosum in mild cognitive impairment and Alzheimer’s disease. Dement Geriatr Cogn Disord 21, 215–220, doi: 10.1159/000090971 (2006). 16415572

[b39] Paul L. K. Developmental malformation of the corpus callosum: a review of typical callosal development and examples of developmental disorders with callosal involvement. J Neurodev Disord 3, 3–27, doi: 10.1007/s11689-010-9059-y (2011). 21484594 PMC3163989

[b40] Zhou J., Gennatas E. D., Kramer J. H., Miller B. L. & Seeley W. W. Predicting regional neurodegeneration from the healthy brain functional connectome. Neuron 73, 1216–1227 (2012). 22445348 10.1016/j.neuron.2012.03.004 PMC3361461

[b41] Buckner R. L. *et al.* Molecular, structural, and functional characterization of Alzheimer’s disease: evidence for a relationship between default activity, amyloid, and memory. J Neurosci 25, 7709–7717, doi: 10.1523/jneurosci.2177-05.2005 (2005). 16120771 PMC6725245

[b42] Scahill R. I., Schott J. M., Stevens J. M., Rossor M. N. & Fox N. C. Mapping the evolution of regional atrophy in Alzheimer’s disease: unbiased analysis of fluid-registered serial MRI. Proc Natl Acad Sci USA 99, 4703–4707, doi: 10.1073/pnas.052587399 (2002). 11930016 PMC123711

[b43] Iturria-Medina Y., Sotero R. C., Toussaint P. J., Evans A. C. & Initiative A. s. D. N. Epidemic spreading model to characterize misfolded proteins propagation in aging and associated neurodegenerative disorders. PLoS Comput Biol 10, e1003956, doi: 10.1371/journal.pcbi.1003956 (2014). 25412207 PMC4238950

[b44] Braak H. & Braak E. Neuropathological stageing of Alzheimer-related changes. Acta Neuropathol 82, 239–259 (1991). 1759558 10.1007/BF00308809

[b45] Whitwell J. L. *et al.* 3D maps from multiple MRI illustrate changing atrophy patterns as subjects progress from mild cognitive impairment to Alzheimer’s disease. Brain 130, 1777–1786, doi: 10.1093/brain/awm112 (2007). 17533169 PMC2752411

[b46] Drzezga A. *et al.* Cerebral glucose metabolism in patients with AD and different APOE genotypes. Neurology 64, 102–107, doi: 10.1212/01.WNL.0000148478.39691.D3 (2005). 15642911

[b47] Brier M. R. *et al.* Loss of intranetwork and internetwork resting state functional connections with Alzheimer’s disease progression. J Neurosci 32, 8890–8899, doi: 10.1523/JNEUROSCI.5698-11.2012 (2012). 22745490 PMC3458508

[b48] Kluger A., Gianutsos J. G., Golomb J., Ferris S. H. & Reisberg B. Motor/psychomotor dysfunction in normal aging, mild cognitive decline, and early Alzheimer’s disease: diagnostic and differential diagnostic features. Int Psychogeriatr 9 Suppl 1, 307–316; discussion 317-321 (1997). 9447451 10.1017/s1041610297005048

[b49] Ghilardi M. F. *et al.* Visual feedback has differential effects on reaching movements in Parkinson’s and Alzheimer’s disease. Brain Res 876, 112–123 (2000). 10973599 10.1016/s0006-8993(00)02635-4

[b50] Kensinger E. A., Shearer D. K., Locascio J. J., Growdon J. H. & Corkin S. Working memory in mild Alzheimer’s disease and early Parkinson’s disease. Neuropsychology 17, 230–239 (2003). 12803428 10.1037/0894-4105.17.2.230

[b51] Canuet L. *et al.* Network Disruption and Cerebrospinal Fluid Amyloid-Beta and Phospho-Tau Levels in Mild Cognitive Impairment. The Journal of Neuroscience 35, 10325–10330 (2015). 26180207 10.1523/JNEUROSCI.0704-15.2015 PMC6605340

[b52] Mintun M. *et al.* [11C] PIB in a nondemented population Potential antecedent marker of Alzheimer disease. Neurology 67, 446–452 (2006). 16894106 10.1212/01.wnl.0000228230.26044.a4

[b53] Daselaar S. M. *et al.* Less wiring, more firing: low-performing older adults compensate for impaired white matter with greater neural activity. Cerebral cortex 25, 983–990, doi: 10.1093/cercor/bht289 (2015). 24152545 PMC4366614

[b54] Reuter-Lorenz P. A. & Cappell K. A. Neurocognitive aging and the compensation hypothesis. Current Directions in Psychological Science 17, 177–182, doi: 10.1111/j.1467-8721.2008.00570.x (2008).

[b55] Song J. *et al.* Age-related reorganizational changes in modularity and functional connectivity of human brain networks. Brain Connectivity 4, 662–676, doi: 10.1089/brain.2014.0286 (2014). 25183440 PMC4238253

[b56] Fernández A. *et al.* Brain oscillatory complexity across the life span. Clinical Neurophysiology: Official Journal of the International Federation of Clinical Neurophysiology 123, 2154–2162, doi: 10.1016/j.clinph.2012.04.025 (2012). 22647457

[b57] Walhovd K. B., Fjell A. M. & Espeseth T. Cognitive decline and brain pathology in aging–need for a dimensional, lifespan and systems vulnerability view. Scandinavian Journal of Psychology 55, 244–254, doi: 10.1111/sjop.12120 (2014). 24730622

[b58] Hermundstad A. M. *et al.* Structural foundations of resting-state and task-based functional connectivity in the human brain. Proc Natl Acad Sci USA 110, 6169–6174, doi: 10.1073/pnas.1219562110 (2013). 23530246 PMC3625268

[b59] van den Heuvel M. P., Mandl R. C., Kahn R. S. & Hulshoff Pol H. E. Functionally linked resting-state networks reflect the underlying structural connectivity architecture of the human brain. Hum Brain Mapp 30, 3127–3141, doi: 10.1002/hbm.20737 (2009). 19235882 PMC6870902

[b60] Chao Y. P. *et al.* Probabilistic topography of human corpus callosum using cytoarchitectural parcellation and high angular resolution diffusion imaging tractography. Hum Brain Mapp 30, 3172–3187, doi: 10.1002/hbm.20739 (2009). 19241418 PMC6871153

[b61] Sporns O. Network attributes for segregation and integration in the human brain. Current Opinion in Neurobiology 23, 162–171, doi: 10.1016/j.conb.2012.11.015 (2013). 23294553

[b62] O’Reilly J. X. *et al.* Causal effect of disconnection lesions on interhemispheric functional connectivity in rhesus monkeys. Proc Natl Acad Sci USA 110, 13982–13987, doi: 10.1073/pnas.1305062110 (2013). 23924609 PMC3752223

[b63] Shen K. *et al.* Stable long-range interhemispheric coordination is supported by direct anatomical projections. Proceedings of the National Academy of Sciences 112, 6473–6478 (2015). 10.1073/pnas.1503436112 PMC4443345 25941372

[b64] Tavor I. *et al.* Task-free MRI predicts individual differences in brain activity during task performance. Science 352, 216–220, doi: 10.1126/science.aad8127 (2016). 27124457 PMC6309730

[b65] Mennes M., Kelly C., Colcombe S., Castellanos F. X. & Milham M. P. The extrinsic and intrinsic functional architectures of the human brain are not equivalent. Cerebral cortex 23, 223–229, doi: 10.1093/cercor/bhs010 (2013). 22298730 PMC3513960

[b66] Musso F., Brinkmeyer J., Mobascher A., Warbrick T. & Winterer G. Spontaneous brain activity and EEG microstates. A novel EEG/fMRI analysis approach to explore resting-state networks. Neuroimage 52, 1149–1161 (2010). 20139014 10.1016/j.neuroimage.2010.01.093

[b67] Ng K. K., Lo J. C., Lim J. K. W., Chee M. W. L. & Zhou J. Reduced functional segregation between the default mode network and the executive control network in healthy older adults: A longitudinal study. Neuroimage 133, 321–330, doi: 10.1016/j.neuroimage.2016.03.029 (2016). 27001500

[b68] Yeo D. *et al.* Pilot validation of a customized neuropsychological battery in elderly Singaporeans. Neurol J South East Asia 2, 123 (1997).

[b69] Hilal S. *et al.* Prevalence of cognitive impairment in Chinese: epidemiology of dementia in Singapore study. J Neurol Neurosurg Psychiatry 84, 686–692, doi: 10.1136/jnnp-2012-304080 (2013). 23385846

[b70] Xu X. *et al.* Validation of the Total Cerebrovascular Disease Burden Scale in a Community Sample. Journal of Alzheimer’s disease: JAD 52, 1021–1028, doi: 10.3233/JAD-160139 (2016). 27079726

[b71] Visser P., Verhey F., Hofman P., Scheltens P. & Jolles J. Medial temporal lobe atrophy predicts Alzheimer’s disease in patients with minor cognitive impairment. Journal of Neurology, Neurosurgery & Psychiatry 72, 491–497 (2002). 10.1136/jnnp.72.4.491 PMC1737837 11909909

[b72] Fischl B. FreeSurfer. Neuroimage 62, 774–781, doi: 10.1016/j.neuroimage.2012.01.021 (2012). 22248573 PMC3685476

[b73] Fischl B. *et al.* Whole brain segmentation: automated labeling of neuroanatomical structures in the human brain. Neuron 33, 341–355 (2002). 11832223 10.1016/s0896-6273(02)00569-x

[b74] Collinson S. L. *et al.* Corpus callosum morphology in first-episode and chronic schizophrenia: combined magnetic resonance and diffusion tensor imaging study of Chinese Singaporean patients. Br J Psychiatry 204, 55–60, doi: 10.1192/bjp.bp.113.127886 (2014). 24202961

[b75] Fischl B. *et al.* Automatically parcellating the human cerebral cortex. Cerebral cortex 14, 11–22 (2004). 14654453 10.1093/cercor/bhg087

[b76] Klauser P. *et al.* Lack of Evidence for Regional Brain Volume or Cortical Thickness Abnormalities in Youths at Clinical High Risk for Psychosis: Findings From the Longitudinal Youth at Risk Study. Schizophr Bull 41, 1285–1293, doi: 10.1093/schbul/sbv012 (2015). 25745033 PMC4601700

[b77] Yan C. G. & Zang Y. F. DPARSF: A MATLAB Toolbox for “Pipeline” Data Analysis of Resting-State fMRI. Front Syst Neurosci 4, 13, doi: 10.3389/fnsys.2010.00013 (2010). 20577591 PMC2889691

[b78] Qiu Y.-w. *et al.* Short-term UROD treatment on cerebral function in codeine-containing cough syrups dependent male individuals. European radiology, 1–10 (2015). 10.1007/s00330-015-4139-8 26662031

[b79] Qiu Y.-W. *et al.* Regional Homogeneity Changes in Heroin-dependent Individuals: Resting-State Functional MR Imaging Study. Radiology 261, 551–559, doi: 10.1148/radiol.11102466 (2011). 21875854

[b80] Yan C. G., Craddock R. C., He Y. & Milham M. P. Addressing head motion dependencies for small-world topologies in functional connectomics. Front Hum Neurosci 7, 910, doi: 10.3389/fnhum.2013.00910 (2013). 24421764 PMC3872728

[b81] Zuo X. N. *et al.* Growing together and growing apart: regional and sex differences in the lifespan developmental trajectories of functional homotopy. J Neurosci 30, 15034–15043, doi: 10.1523/JNEUROSCI.2612-10.2010 (2010). 21068309 PMC2997358

[b82] Brett M., Penny W. & Kiebel S. Introduction to random field theory. Vol. Human Brain Function (2nd edition) (Elsevier Academic Press, 2003).

[b83] Song X. W. *et al.* REST: a toolkit for resting-state functional magnetic resonance imaging data processing. PLoS One 6, e25031, doi: 10.1371/journal.pone.0025031 (2011). 21949842 PMC3176805

[b84] Mattsson N. *et al.* Brain structure and function as mediators of the effects of amyloid on memory. Neurology 84, 1136–1144 (2015). 25681451 10.1212/WNL.0000000000001375 PMC4371407

[b85] Jenkinson M., Bannister P., Brady M. & Smith S. Improved optimization for the robust and accurate linear registration and motion correction of brain images. Neuroimage 17, 825–841 (2002). 12377157 10.1016/s1053-8119(02)91132-8

