# Supplementary material for: Inter-hemispheric functional dysconnectivity mediates the association of corpus callosum degeneration with memory impairment in AD and amnestic MCI
Source: Sci Rep. 2016 Sep 1;6:32573. doi: 10.1038/srep32573 (PMC5007647; doi:10.1038/srep32573)
Supplement: Supplementary Information [file srep32573-s1.docx]

**Title: Inter-hemispheric functional dysconnectivity mediates the association of corpus callosum degeneration with memory impairment in AD and amnestic MCI**

**Running title: Corpus callosum and functional homotopy reductions in AD and amnestic MCI**

**Authors:** Yingwei Qiu, Siwei Liu, Saima Hilal, Yng Miin Loke, Mohammad Kamran Ikram, Xu Xin, Tan Boon Yeow, Narayanaswamy Venketasubramanian, Christopher Li-Hsian Chen, Juan Zhou

**SUPPLEMENTARY MATERIALS**

**Supplementary Methods**

*Participant selection criterions*

Out of 411 recruited participants, 10 were excluded for not having MRI data and 193 participants were not included due to presence of cerebral vascular disease (CeVD, see below for the CeVD definition) on MRI scans and with Vascular Dementia (VaD) diagnosis. Cerebrovascular Disease (CeVD) was defined as the presence of any of the following: 1) cortical infarcts; 2) two or more lacunes; 3) confluent white matter hyperintensities (WMH) (in two regions of the brain (Age Related WM Changes scale score ≥ 8)^1^. Individuals with significant CeVD were excluded from the analysis. We also excluded 27 participants due to excessive head motion in task free fMRI data or motion artifacts in 3D-T1 WI data (see details in Image Preprocessing). Finally, 11 subjects were excluded due to absence of subjective cognitive complaints and an additional 22 with no impairment in memory domain on objective neuropsychological testing. The final sample consisted of 66 healthy controls (Ctrl, 39 female, 52-80 years old), 41 amnestic MCI patients (aMCI, 22 female, 50-84 years old) and 41 AD patients (25 female, 55-91 years old). Each participant underwent extensive clinical and neuropsychological evaluation, including the Clinical Dementia Rating Scale (CDR)^2^, the Mini-Mental State Examination (MMSE)^3^, the Montreal Cognitive Assessment (MoCA)^4^ and a standard neuropsychological battery (see details in our previous work)^5,6^.

*Neuropsychological measurements and diagnoses*

The detailed neuropsychological assessments include seven domains, five of which are non-memory domains. The non-memory domains included the following: (1) executive function (frontal assessment battery^7^ and maze task^8^; (2) attention (digit span, visual memory span^9^ and auditory detection tests^10^); (3) language (Boston naming test^11^ and verbal fluency^12^; (4) visuomotor speed (symbol digit modality test^13^ and digit cancellation^14^; (5) visuoconstruction (the Wechsler memory scale revised visual reproduction copy task^9^, clock drawing^15^ and the Wechsler adult intelligence scale-revised subtest of block design^16^. The memory domains included: verbal memory (word list recall^17^ and story recall) and visual memory (picture recall and the Wechsler memory scale-revised visual reproduction^9^. The assessment was administered according to the subject’s habitual language and was completed in approximately one hour.

Z-scores were then derived for individual subtests. All z-scores were adapted so that a greater value reflects better performance. Z-scores for individual domains were computed by summing up the Z-scores of each subtest and dividing by the number of the subtests under that domain. Domain specific z-scores were used to compute the final global cognitive composite score. The visual and verbal memory scores were combined into a composite memory score. Only 133 subjects (33 AD, 41 aMCI and 59 controls) who completed all the tasks were included in the statistical analysis of cognition.

MCI was defined as subjects with subjective cognitive complains and impairment in memory domain on the neuropsychological test battery. The etiological diagnosis of AD were made using the National Institute of Neurological and Communicative Disorders and Stroke and the Alzheimer's Disease and Related Disorders Association (NINCDS-ADRDA)^18^.

*Imaging acquisition*

High-resolution T1-weighted structural MRI was acquired using MPRAGE (magnetization-prepared rapid gradient echo) sequence (192 continuous sagittal slices, TR/TE/TI = 2300/1.9/900 ms, flip angle = 9˚, FOV = 256 × 256 mm2, matrix = 256 × 256, isotropic voxel size = 1.0 × 1.0 × 1.0 mm^3^, bandwidth = 240 Hz/pixel). A task-free fMRI was acquired using a single-shot EPI sequence (TR/TE = 2300/25 ms, flip angle = 90˚, FOV= 192 ×192 mm, matrix = 64 × 64, voxel size = 3.0 × 3.0 × 3.0 mm^3^, 128 volumes).

*Corpus callosum volume calculation*

Prior to processing, all scans were visually examined for motion artifacts or other distortions by a trained rater, and only scans with no visible distortion were included in the sample. The automated procedures for subcortical volume measurements of different brain structures have been described previously. Briefly, this process includes motion correction, removal of non-brain tissue using a hybrid watershed/surface deformation procedure^19^, automated Talairach transformation, segmentation of the subcortical white matter and deep gray matter volumetric structures (including the hippocampus, amygdala, caudate, putamen, and ventricles)^20,21^, intensity normalization, tessellation of the gray-white matter boundary, automated topology correction^22^ and surface deformation following intensity gradients to optimally place the gray-white matter and gray matter/CSF borders at the location where the greatest shift in intensity defines the transition to the other tissue class.

*Task-free fMRI data preprocessing*

The first 5 images were discarded to allow for signal stabilization and subject adaptation. The remaining images were first corrected for slice time differences and head motion. We then co-registered the individual functional images to T1-weighted MR images. The T1-weighted MR images were segmented (gray matter, white matter, and cerebrospinal fluid) and normalized to the standard structural MRI template in the Montreal Neurologic Institute space using a 12-parameter nonlinear transformation. These transformation parameters were applied to the functional images. To remove the sources of possible spurious variance from each voxel’s fMRI time series, we performed the following: (a) removed linear trends; (b) regressed out nuisance signals (white matter, cerebrospinal fluid signals, and six head-motion parameters); c) performed spikes removal; and (d) applied temporal bandpass filtering (0.01–0.08 Hz).

To account for the differences in the geometric configuration of the cerebral hemispheres, we further transformed the preprocessed functional images to a symmetric space following a previous approach^23^. To achieve this, we used the following procedure: (a) the normalized gray matter images were averaged for all participants to create a group-specific gray matter template; (b) the group-specific gray matter template was then averaged with its left-right flipped version to generate a group-specific symmetrical gray matter template; (c) normalized subject-specific gray matter images to the group-level symmetrical gray matter template; d) applied the resulting nonlinear transformations to convert subject-specific fMRI data into group-level symmetrical gray matter template space; e) re-sampled the fMRI data in the symmetrical space at a resolution of 3 × 3 × 3 mm^3^; and f) spatially smoothed fMRI data with a 6-mm full-width at half-maximum isotropic Gaussian kernel.

**Supplementary Results**

*Validation analysis: Age-matched and right-handers only*

To ensure that the observed group differences in inter-hemispheric homotopic functional connectivity were not confounded by age difference, we repeated the analysis in an age-matched sub-cohort of all subjects (AD=32, aMCI=38, control=38). The inter-hemispheric homotopic functional connectivity changes in AD and aMCI remained approximately the same as the primary analysis (Supplementary Fig. 2, Table 2). Inter-hemispheric interaction can vary with strength and consistency of handedness^24^. To remove the potential confounding effect of handedness, we repeated the group analyses on right-handers only (AD = 41, aMCI=38, Control = 61) and found similar patterns (results not shown).

*Validation analysis: group differences in AD, a-CIND and healthy control*

We also repeated the analysis by including the subjects with cognitive impairment (memory) on neuropsychological assessment but without subjective complaint on memory in case (amnestic cognitive impairment no dementia, a-CIND), the results are also similar to the primary findings (Supplementary Fig. 4, Table 3).

*Mediation effects of CC subregions on memory deficits by inter-hemispheric functional connectivity*

In addition to the total CC, inter-hemispheric functional connectivity also partially (27.5-36.4%) mediated the effects of CC subregions, including CC2, CC3, CC4 and CC5, on memory in AD and aMCI patients (Supplementary Fig. 5). Notably, such mediation effects of CC3 and CC5 subregions on memory deficits by inter-hemispheric functional connectivity remained after additionally controlling for hippocampal volume (CC3 (direct effect = 0.72, indirect effect = 0.25) and CC5 (direct effect = 0.64, indirect effect = 0.19)).

**Supplementary Table 1. Differences in corpus callosum subregions volumes between healthy control, MCI due to AD-intermediate likelihood and AD fulfilled research criterion.** Values represent the mean ± standard deviation. The last column represents the p-values of Analysis of covariance (ANCOVA) between groups; ‘*’ indicates significant differences between the three groups at the threshold of p < 0.05. Superscript letters indicate whether group mean was significantly worse than healthy control (c) or subjects with amnestic mild cognitive impairment (m) based on post-hoc pairwise comparisons (p < 0.05).

|  | **Ctrl (n=54)** | **MCI (n=18)** | **AD (n=37)** | **P values** |
| --- | --- | --- | --- | --- |
| **CC1 (mm^3^)** | 686.2 (109.0) | 592.7 (170.4) | 533.4 (85.1) ^c^ | <0.001* |
| **CC2 (mm^3^)** | 358.3 (70.8) | 283.9 (68.3) ^c^ | 250.9 (44.0) ^c^ | <0.001* |
| **CC3 (mm^3^)** | 342.7 (60.9) | 288.2 (58.2) ^c^ | 249.8 (42.4) ^mc^ | <0.001* |
| **CC4 (mm^3^)** | 307.7 (66.3) | 260.6 (53.7) ^c^ | 219.0 (50.7) ^mc^ | <0.001* |
| **CC5 (mm^3^)** | 861.9 (123.3) | 758.4 (140.3) ^c^ | 686.8 (96.4) ^c^ | <0.001* |
| **CCtotal (mm^3^)** | 2556.7 (347.8) | 2245.9 (486.4) ^c^ | 1940.1 (266.1) ^mc^ | <0.001* |

Abbreviations: Ctrl, Control; MCI, mild cognitive impairment; AD, Alzheimer’s disease; CC, corpus callosum.

**Supplementary Table 2. Group differences in the inter-hemispheric functional connectivity between healthy controls, aMCI and AD.** All results were reported at a height threshold of p<0.01 and cluster threshold of p<0.05 with GRF correction.

| **Regions** | **Brodmann Areas** | **MNI Coordinates** | | | **Peak**  **t-score** | **Cluster size**  **(mm ^3^ )** |
| --- | --- | --- | --- | --- | --- | --- |
|  |  | **X** | **Y** | **Z** |  |  |
| **ANCOVA** | | | | | | |
| STG/IPL/SMG/INS/ROL | 13,22,40,41 | 57 | -30 | 18 | 14.3573 | 562 |
| MFG | 10,46 | 39 | 48 | 18 | 9.5899 | 155 |
| PoCG/PreCG | 1,2,3,4,5,6 | 30 | -36 | 69 | 9.8883 | 211 |
| PreC/PCC/CAL | 7,23,30,31 | 27 | -66 | 30 | 8.6489 | 87 |
|  |  |  |  |  |  |  |
| **AD < Control** | | | | | | |
| PoCG /SPG/PreC/PreCG/MOG | 3,4,6,7,18,19 | 30 | -36 | 69 | -5.3547 | 1897 |
| STG/MTG/ROL/SMG/IPL | 6,22,40,42 | 54 | -33 | 15 | -4.7737 | 1222 |
| MFG/SFG/DLPFC | 10,46 | 33 | 33 | 18 | -4.2719 | 198 |
| MTG/ PUT/ PHG | 28,36,38 | 21 | 12 | -42 | -4.1962 | 196 |
|  |  |  |  |  |  |  |
| **AD < aMCI** | | | | | | |
| STG/IPL/MTG/SMG/ROL | 13,22,39,40,41 | 42 | -21 | 18 | -4.9586 | 1051 |
| MFG/ IFG/SFG | 9,10,46 | 39 | 51 | 18 | -4.4512 | 190 |

Abbreviations: AD, Alzheimer's disease; aMCI, amnestic cognitive impairment no dementia; AAL, Anatomical Automatic Labeling; MNI, Montréal Neurological Institute; STG, superior temporal gyrus; MTG, Middle temporal gyurs; INS, Insula; SMG, Supramarginal gyrus; ROL, Rolandic; MFG, middle temporal gyrus; PoCG, postcentral gyrus; PreCG, Precentral gyrus; PreC, Precuneus; CAL, Calcarine; SPG, Superior parietal gyrus; STG, superior temporal gyrus; DLPFC, Dorsolateral prefrontal cortex; PUT, Putamen; PHG, Parahippocampal gyrus; IFG, Inferior frontal gyrus; MOG, middle occipital gyrus; GRF, Gaussian random field.

**Supplementary Table 3. Regions showing inter-hemispheric functional connectivity differences between the three groups in the age-matched sub cohort.** Whole-brain voxelwise ANCOVA analyses were performed on homotopic functional connectivity across 32 AD, 38 aMCI and 38 controls. Results were reported at a height threshold of p<0.01 and cluster threshold of p<0.05 with GRF correction.

| **Brain regions**  **(AAL)** | **Brodmann**  **Areas** | **MNI Coordinates** | | | **Peak**  **t-score** | **Cluster Size**  **(mm^3^ )** |
| --- | --- | --- | --- | --- | --- | --- |
|  |  | **X** | **Y** | **Z** |  |  |
|  |  |  |  |  |  |  |
| **ANCOVA** | | | | | | |
| STG/MTG/IPL | 13,22,40,41,42 | 57 | -30 | 15 | 14.5285 | 289 |
| MOG/CUN/SOG | 18,19 | 33 | -87 | -3 | 9.1463 | 162 |
| CAL/PreC/PCC | 23,31 | 9 | -63 | 18 | 8.9011 | 143 |
| SFG/MFG | 9,10,46 | 42 | 51 | 18 | 10.2103 | 159 |
| PreC/PoCG | 4,5,7 | 9 | -51 | 66 | 9.1895 | 74 |
|  |  |  |  |  |  |  |
| **AD < Control** | | | | | | |
| PoCG/PreCG/SMA/MFG | 3,4,6,7,40 | 3 | -9 | 75 | -4.5363 | 1099 |
| STG/ROL/MTG/IPL | 18,19,22,31 | 48 | -36 | 24 | -4.8319 | 1782 |
| MFG/SFG | 10,46 | 36 | 27 | 21 | -4.2609 | 197 |
|  |  |  |  |  |  |  |
| **AD < aMCI** | | | | | | |
| STG/MTG/IPL/ROL/INS | 13,22,40,41 | 57 | -30 | 18 | -5.1381 | 558 |
| MFG/SFG | 9,10,46 | 39 | 51 | 18 | -4.4679 | 323 |
| CUN/SOG | 18,19 | 15 | -96 | 21 | -3.634 | 138 |
| CAL/PreC/PCC | 7,23,31 | 9 | -63 | 18 | -4.122 | 172 |

Abbreviations: AD, Alzheimer's disease; a-MCI, amnestic cognitive impairment no dementia; AAL, Anatomical Automatic Labeling; MNI, Montréal Neurological Institute; ROL, Rolandic; STG, Superior temporal gyrus; MTG, Middle temporal gyrus; SFG, Superior frontal gyrus; PreC, Precuneus; PoCG, Postcentral gyrus; PreCG, Precentral gyrus; SMA, Supplement motor area; IPL, Inferior parietal lobe; MFG, Middle temporal gyrus; SFG, Superior frontal gyrus; SOG, Superior occipital gyrus; MFG, Middle frontal gyrus; SPL, Superior parietal lobe; CAL, Calcarine; CL, Claustrum; INS, Insular; PCC, Posterior cingulate cortex.

**Supplementary Table 4. Group differences in the inter-hemispheric homotpic functional connectivity between healthy controls, a-CIND and AD.** Whole-brain voxelwise ANCOVA analyses were performed on homotopic inter-hemispheric functional connectivity across 41 AD, 52 a-CIND and 66 controls. Results were reported at a height threshold of p<0.01 and cluster threshold of p<0.05 with GRF correction.

| **Regions** | **Brodmann**  **Areas** | **MNI Coordinates** | | | **Peak**  **t-score** | **Cluster size**  **(mm^3^ )** |
| --- | --- | --- | --- | --- | --- | --- |
|  |  | **X** | **Y** | **Z** |  |  |
| **ANCOVA** | | | | | | |
| STG/MTG/SMG/ROL | 13,22,40,41 | 54 | -18 | 9 | 14.8117 | 741 |
| MFG | 10,46 | 39 | 51 | 18 | 11.264 | 153 |
| PoCG/PreCG | 1,2,3,4,5,6 | 30 | -36 | 69 | 9.8883 | 95 |
| PreC/CAL | 7,23,30,31 | 6 | -63 | 18 | 9.7045 | 90 |
|  |  |  |  |  |  |  |
| **AD < Control** | | | | | | |
| PoCG /SPG/PreC/PreCG | 3,4,6,7,18,19 | 30 | -36 | 69 | -5.3547 | 1897 |
| STG/MTG/ROL/SMG | 6,22,40,42 | 54 | -33 | 15 | -4.7737 | 1222 |
| MFG | 10,46 | 33 | 33 | 18 | -4.2719 | 198 |
| MTG/ PUT/ PHG | 28,36,38 | 21 | 12 | -42 | -4.1962 | 196 |
|  |  |  |  |  |  |  |
| **AD < aCIND** | | | | | | |
| STG/MTG/SMG/ROL | 7,13,22,40,41 | 54 | -18 | 9 | -5.5312 | 1886 |
| PreC/CUN/ CAL | 18,19,23,30,31 | 6 | -63 | 18 | -4.557 | 383 |
| MFG/ IFG | 10,46 | 39 | 51 | 18 | -4.7829 | 329 |

Abbreviations: AD, Alzheimer's disease; a-CIND, amnestic cognitive impairment no dementia; AAL, Anatomical Automatic Labeling; MNI, Montréal Neurological Institute; STG, superior temporal gyrus; MTG, Middle temporal gyurs; SMG, Supramarginal gyrus; ROL, Rolandic; MFG, middle temporal gyrus; PoCG, postcentral gyrus; PreCG, Precentral gyrus; PreC, Precuneus; CAL, Calcarine; SPG, Superior parietal gyrus; STG, superior temporal gyrus; PUT, Putamen; PHG, Parahippocampal gyrus; IFG, Inferior frontal gyrus; GRF, Gaussian random field.

**Supplementary Table 5. CC degeneration is associated with decreased inter-hemispheric homotopic functional connectivity in AD and aMCI patients.** Regions whose homotopic inter-hemispheric functional connectivity showed significant correlations with volume of CC and its sub-regions. Results were reported at a height threshold of p<0.01 and cluster threshold of p<0.05 with GRF correction.

| **Regions** | **Brodmann**  **Areas** | **MNI Coordinates** | | | **Peak**  **t-score** | **Cluster Size (mm^3^)** |
| --- | --- | --- | --- | --- | --- | --- |
|  |  | **X** | **Y** | **Z** |  |  |
| *CC2* | | | | | | |
| IOG/CUN/PreC/CAL/PCC | 7,18,19,30 | 27 | -69 | 48 | 0.43212 | 513 |
| MCC/SMA/SFG/MFG | 6,24,32 | 3 | 6 | 39 | 0.43809 | 205 |
| *CC3* |  |  |  |  |  |  |
| IOG/CUN/LING/MOG | 17,18,19 | 33 | -87 | -9 | 0.47212 | 895 |
| PoCG/SFG | 3,4,6 | 30 | -36 | 69 | 0.39599 | 179 |
| *CC4* |  |  |  |  |  |  |
| MTG/STG/ROL/SMG | 22,39,40,41,42 | 57 | -57 | 9 | 0.49067 | 463 |
| *CC5* |  |  |  |  |  |  |
| CAL/CUN/LING/SOG | 17,18,19 | 9 | -87 | 0 | 0.39253 | 225 |
| MTG/STG/ROL/SMG/IPL | 12,22,40,43 | 57 | -51 | 6 | 0.49240 | 904 |
| MFG/SFG | 8,9,10,32 | 24 | 24 | 45 | 0.41595 | 165 |
| *CC total* |  |  |  |  |  |  |
| MTG/STG/ROL/SMG | 21,22,40,41 | 57 | -51 | 6 | 0.50929 | 584 |
| CUN/LING/CAL/IOG | 7,17,18,19 | 12 | -87 | 27 | 0.41473 | 411 |
| SFG/MFG | 8,9,10 | 21 | 48 | 48 | 0.39183 | 175 |

Abbreviations: AD, Alzheimer's disease; aMCI, amnestic cognitive impairment no dementia; AAL, Anatomical Automatic Labeling; MNI, Montréal Neurological Institute; MCC, Middle cingulate cortex; MFG, Middle frontal gyrus; MTG, Middle temporal gyrus; STG, Superior temporal gyrus; ROL, Rolandic; SOG, Superior occipital gyrus; MOG, Middle occipital gyrus; SMA, Supplement motor area; SFG, Superior frontal gyrus; MFG, Middle frontal gyrus; IOG, Inferior occipital gyrus; SPL, Superior parietal lobe; IPL, Inferior parietal lobe; ITG, Inferior temporal gyrus; CUN, Cuneus; CAL, Calcarine; STG, superior temporal gyrus; LING, Lingual gyrus; SMG, SupraMarginal gyrus. PoCG, postcentral gyrus.

**Supplementary Table 6. CC degeneration is associated with decreased inter-hemispheric homotopic functional connectivity in MCI due to AD-intermediate likelihood and AD patients fulfilled research diagnosis. Regions whose homotopic inter-hemispheric functional connectivity showed significant correlations with volume of CC and its sub-regions. Results were reported at a height threshold of p<0.05 and cluster threshold of p<0.05 with GRF correction.**

| **Regions** | **Brodmann**  **Areas** | **MNI Coordinates** | | | **Peak**  **t-score** | **Cluster Size (mm^3^ )** |
| --- | --- | --- | --- | --- | --- | --- |
|  |  | **X** | **Y** | **Z** |  |  |
| CC1 |  |  |  |  |  |  |
| MFG/SFG/ACC/MCC/SMA | 6,8,9,10,32 | 33 | 39 | 42 | 0.57432 | 1625 |
| STG/ROL/IFG/PoCG | 22,41,44 | 60 | 15 | 6 | 0.42101 | 521 |
| PreC/SPL/IPL/MOG/MTG/CUN | 7,19,31,39 | 45 | -63 | 54 | 0.56034 | 993 |
| *CC2* | | | | | | |
| SFG/MFG/MCC/SMA | 6,8,9,24,32 | 18 | 24 | 45 | 0.47267 | 817 |
|  |  |  |  |  |  |  |
| *CC3* |  |  |  |  |  |  |
| IOG/CUN/LING/MOG | 17,18,19,37 | 33 | -87 | -9 | 0.50677 | 905 |
| PreC/IPL/SPL/SOG/CUN/MTG | 7,19,31,40 | 3 | -63 | 24 | 0.46689 | 878 |
| MFG/SFG/MCC | 8,9,10,32 | 36 | 24 | 21 | 0.52259 | 1101 |
|  |  |  |  |  |  |  |
| *CC5* |  |  |  |  |  |  |
| MTG/STG/IPL/ROL/SOG/MFG | 7,819,22,40 | 33 | -81 | 36 | 0.51374 | 2063 |
|  |  |  |  |  |  |  |
| *CC total* |  |  |  |  |  |  |
| PreC/IPL/SPL/CAL | 7,23,30,31,40 | 24 | -72 | 51 | 0.4387 | 594 |
| SFG/MFG/MCC | 6,8,9,10,32 | 21 | 24 | 45 | 0.5256 | 760 |

Abbreviations: AD, Alzheimer's disease; aMCI, amnestic cognitive impairment no dementia; AAL, Anatomical Automatic Labeling; MNI, Montréal Neurological Institute; ACC, Anterior cingulate cortex; MCC, Middle cingulate cortex; MFG, Middle frontal gyrus; MTG, Middle temporal gyrus; STG, Superior temporal gyrus; ROL, Rolandic; SOG, Superior occipital gyrus; PreC, Precuneus; MOG, Middle occipital gyrus; SMA, Supplement motor area; SFG, Superior frontal gyrus; MFG, Middle frontal gyrus; IOG, Inferior occipital gyrus; SPL, Superior parietal lobe; IPL, Inferior parietal lobe; ITG, Inferior temporal gyrus; CUN, Cuneus; CAL, Calcarine; STG, superior temporal gyrus; LING, Lingual gyrus; SMG, SupraMarginal gyrus. PoCG, postcentral gyrus.

**Supplementary Table 7. Correlations between brain measures and memory in patients with AD and aMCI.**

| **Brain measures** | **Memory** | | **Memory*** | |  |
| --- | --- | --- | --- | --- | --- |
|  | **r value** | **p value** | **r value*** | **p value*** |  |
| **CC (sub)regions** |  |  |  | |  |
| CC2 | 0.419 | < 0.001 | 0.297 | 0.012 |  |
| CC3 | 0.441 | < 0.001 | 0.405 | <0.001 |  |
| CC4 | 0.375 | 0.001 | 0.283 | 0.017 |  |
| CC5 | 0.446 | < 0.001 | 0.397 | < 0.001 |  |
| Total CC | 0.424 | < 0.001 | 0.355 | 0.002 |  |
| **VMHC** |  |  |  | |  |
| PreC | 0.358 | 0.002 | 0.293 | 0.013 |  |
| PoCG | 0.445 | < 0.001 | 0.370 | 0.001 |  |
| ROL | 0.416 | < 0.001 | 0.335 | 0.004 |  |

Total CC and CC2, CC3, CC4, CC5 subregions volumes and aberrant inter-hemispheric homotopic functional connectivity revealed by ANCOVA (Figure 2) correlated with memory performance across all patients (AD and aMCI). Significant correlations were reported at p<0.05 with Bonferroni correction, controlling for age and TIV. ‘*’ denotes the results after including hippocampal volume as additional nuisance variable. Abbreviations: AD, Alzheimer's disease; aMCI, amnestic cognitive impairment no dementia; CC, Corpus callosum; PreC, Precuneus; PoCG, Postcentral gyrus; ROL, Rolandic.

**Supplementary Figure 1. Post-hoc analyses of inter-hemispheric homotopic functional connectivity differences between the three groups.** Based on the regions showing group differences in VMHC (ANOVA analysis, Figure 2), we performed post-hoc analyses on the extracted cluster-mean VMHC values per subject. After controlling for age, sex, race, handedness, and head motion, AD patients had lower inter-hemispheric homotopic functional connectivity in the rMFG, rPreC, rPoCG and the rROL compared to healthy control and a-MCI groups.

**
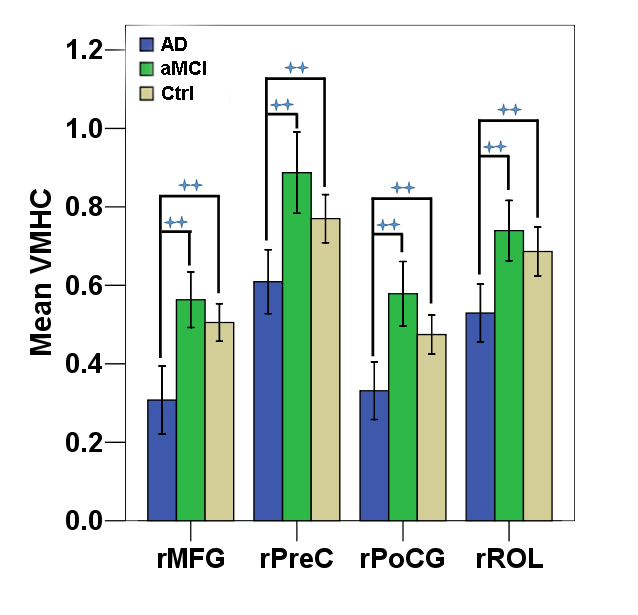
**

p<0.01 with multiple comparison correction

Abbreviations: AD, Alzheimer's disease; a-MCI, amnestic cognitive impairment no dementia; Ctrl, Control; rGM, right brain gray matter; rMFG, right Middle frontal gyrus; rROL, right Rolandic; rPoCG, right postcentral gyrus; rPreC, right Precuneus; VMHC, voxel mirrored homotopic connectivity.

**Supplementary Figure 2. Cross-cohort comparisons of interhemispheric homotopic functional connectivity in the age-matched groups.**


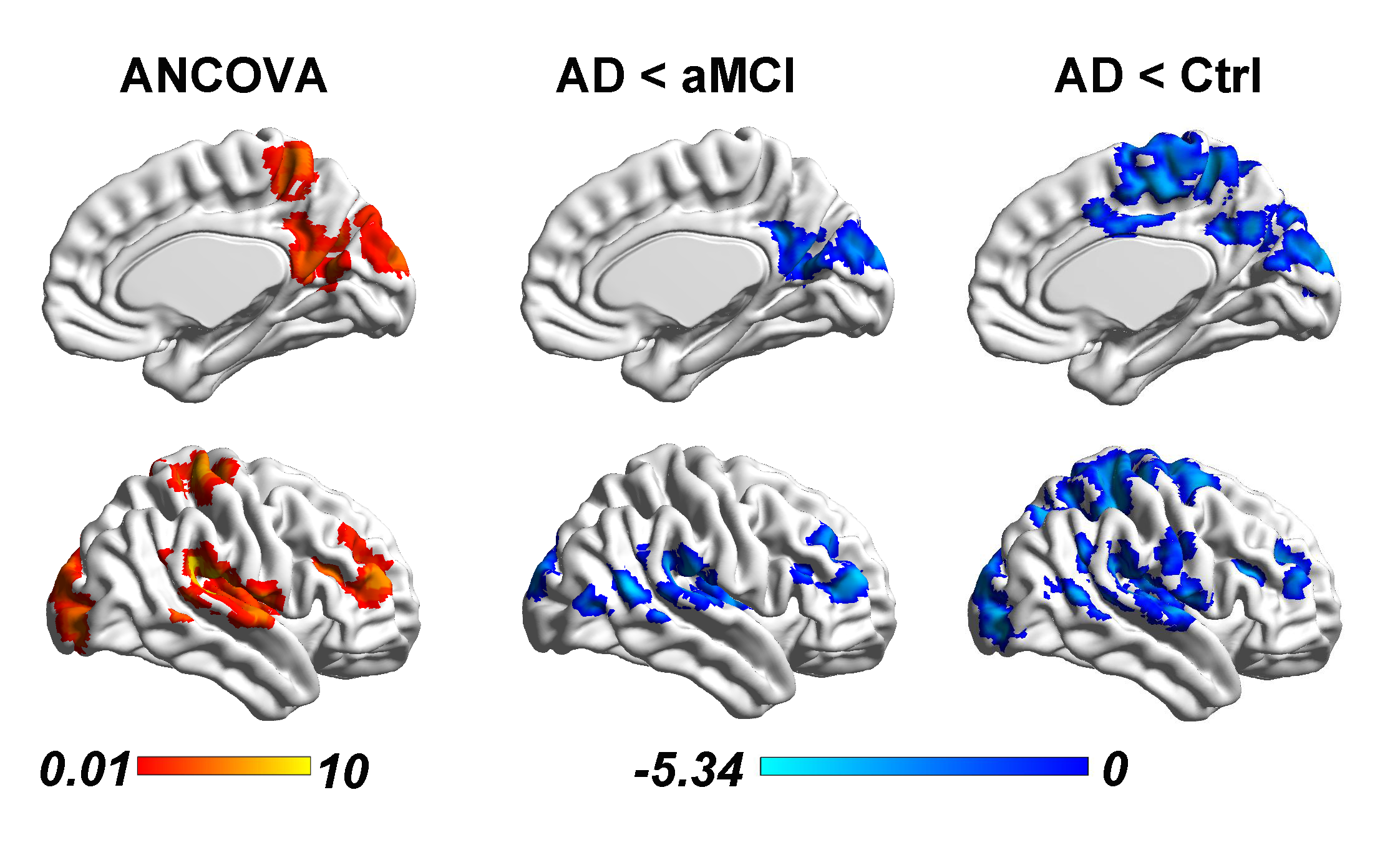


Hot denotes significant different between the three groups. Blue denotes lower VMHC in AD patients and the color bars indicate the T value. Left: ANCOVA showed significant differences in regions of the MFG, PreC, and ROL between the three groups. Middle: AD patients showed decreased VMHC in the MFG, temporal-parietal and occipital regions compared to a-MCI patients. Right: AD patients had decreased VMHC in more widespread brain regions include the MFG, temporal-parietal and occipital regions compared to controls.

Abbreviations: AD, Alzheimer's disease; a-MCI, amnestic cognitive impairment no dementia; Ctrl, Control; MFG, Middle frontal gyrus; ROL, Rolandic; PreC, Precuneus; VMHC, voxel mirrored homotopic connectivity.

**Supplementary Figure 3. Groups differences in inter-hemispheric homotopic functional connectivity between control, MCI due to AD-intermediate likelihood and AD fulfilling research diagnosis.**

**
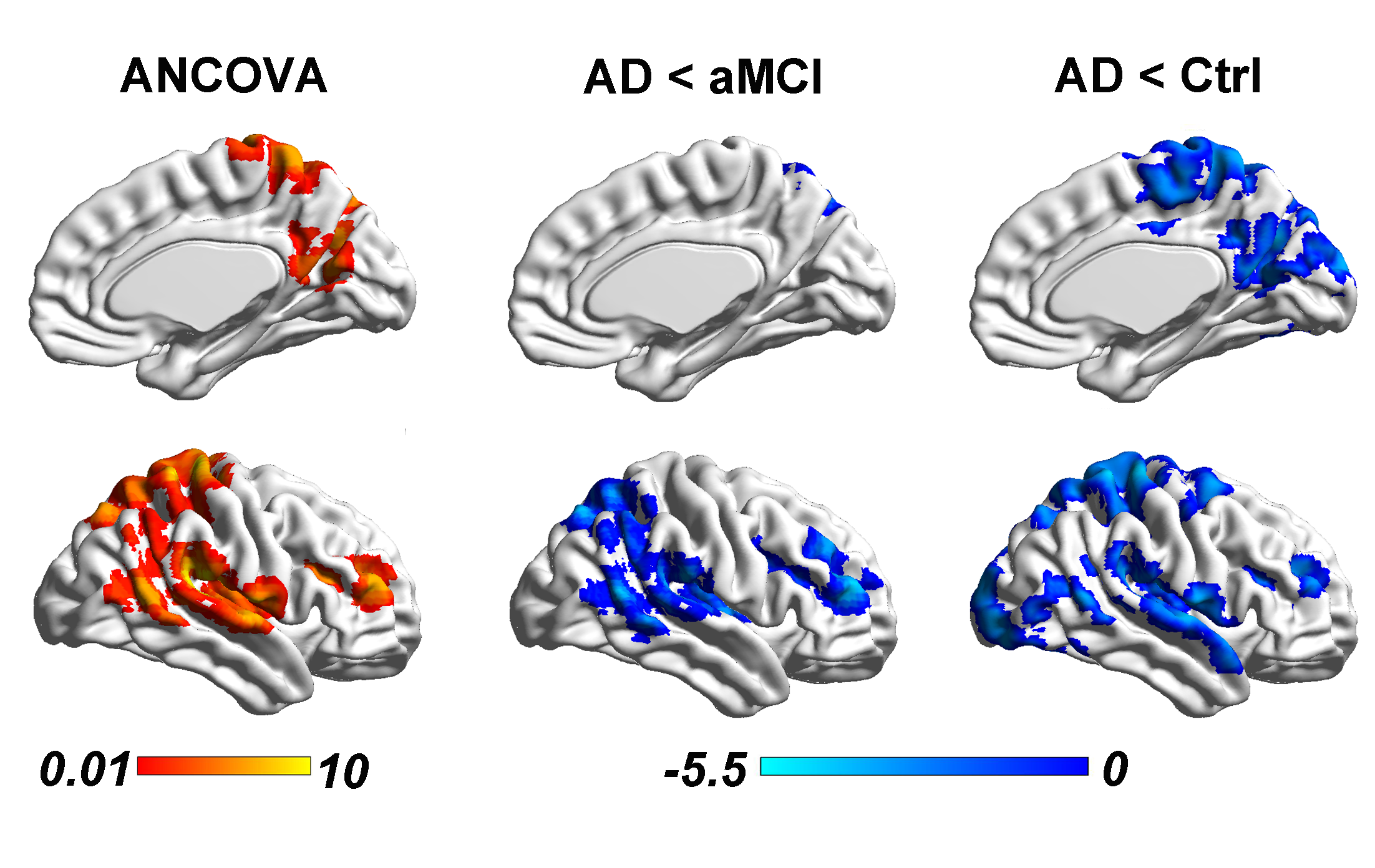
**

Left: After controlling for age, sex, race, handedness, and head motion, the three groups showed significant differences in the inter-hemispheric homotopic functional connectivity (i.e. VMHC) in the MFG, PreC, and ROL (regions highlighted in orange color) using ANOVA (color bar represents F-values). Middle: AD patients showed decreased VMHC in the MFG, temporal-parietal regions compared to MCI due to AD-intermediate likelihood group. Right: AD patients had decreased VMHC in more widespread brain regions include the MFG, temporal-parietal, PreC, and occipital regions compared to controls. All results were reported at a height threshold of p<0.01 and cluster threshold of p<0.05 with GRF correction.

Abbreviations: AD, Alzheimer's disease; aMCI, amnestic cognitive impairment no dementia; Ctrl, Control; MFG, Middle frontal gyrus; ROL, Rolandic; PreC, Precuneus; VMHC, voxel mirrored homotopic connectivity.

**Supplementary Figure** **4**. **Group differences in inter-hemispheric homotopic functional connectivity between control, aCIND and AD.**


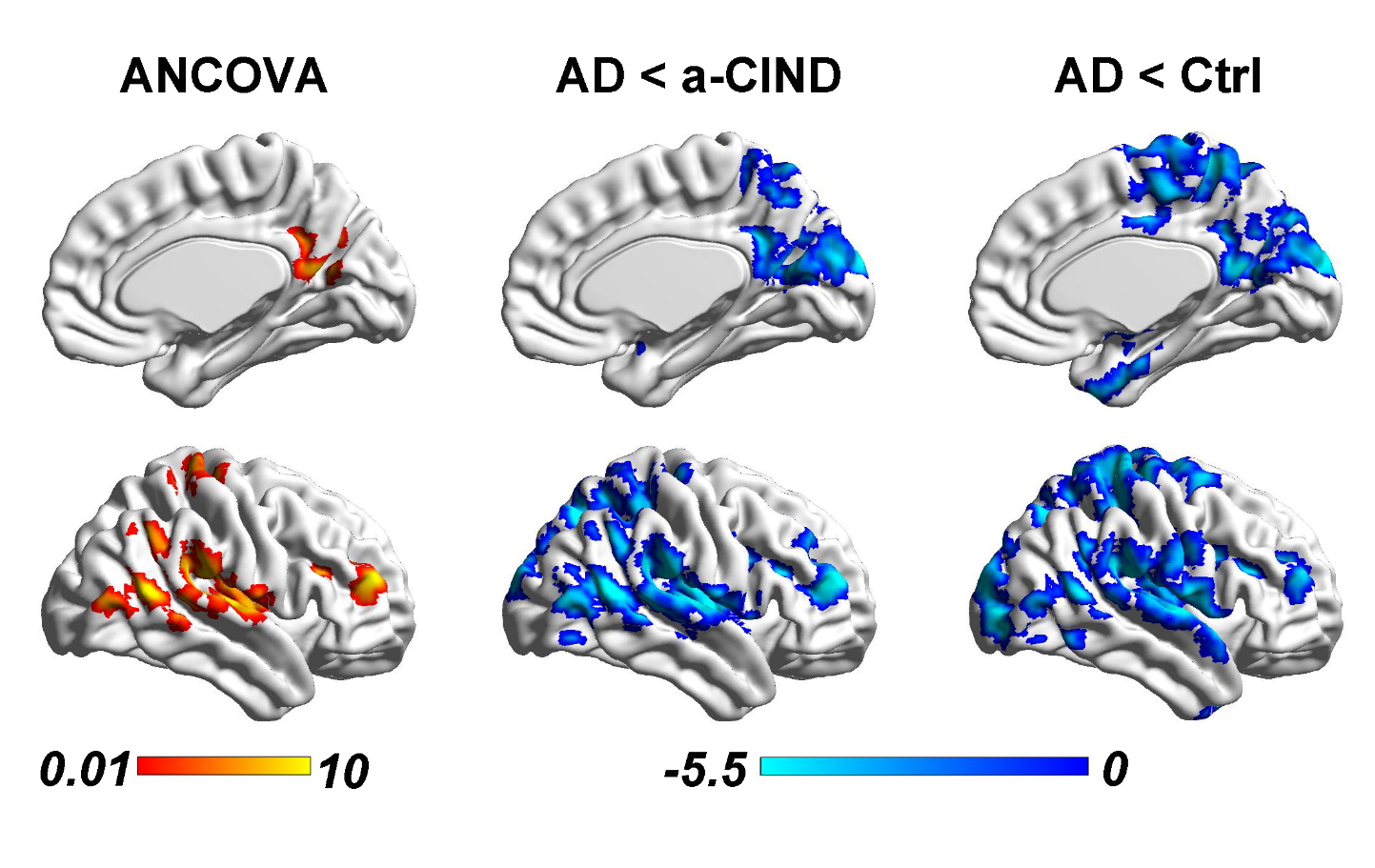


Left: After controlling for age, sex, race, handedness, and head motion, the three groups showed significant differences in the inter-hemispheric homotopic functional connectivity (i.e. VMHC) in the MFG, PreC, PoCG and ROL (regions highlighted in orange color) using ANOVA (color bar represents F-values). Middle: The AD patients had decreased VMHC in the MFG, temporal-parietal and occipital regions (extending to the PCC and insulansula) (regions highlighted in blue color) compared to a-CIND patients. Right: The AD patients had decreased VMHC in more widespread brain regions, including the MFG, temporal-parietal and occipital regions (extending to the PCC, insula and hippocampus), than the controls. The blue color bars indicate t-values of each comparison. No differences in VMHC were detected between a-CIND and controls. All results were reported at a height threshold of p<0.01 and cluster threshold of p<0.05 with GRF correction.

Abbreviations: AD, Alzheimer's disease; a-CIND, amnestic cognitive impairment no dementia; Ctrl, Control; MFG, Middle frontal gyrus; ROL, Rolandic; PoCG, Postcentral gyrus; PreC, Precuneus; PCC, Posterior cingulate cortex; VMHC, voxel mirrored homotopic connectivity; GRF, Gaussian random field.

**Supplementary Figure 5. Inter-hemispheric homotopic functional connectivity mediated the association of corpus callosum degeneration with memory impairment in AD and aMCI after controlling for hippocampal volume.**


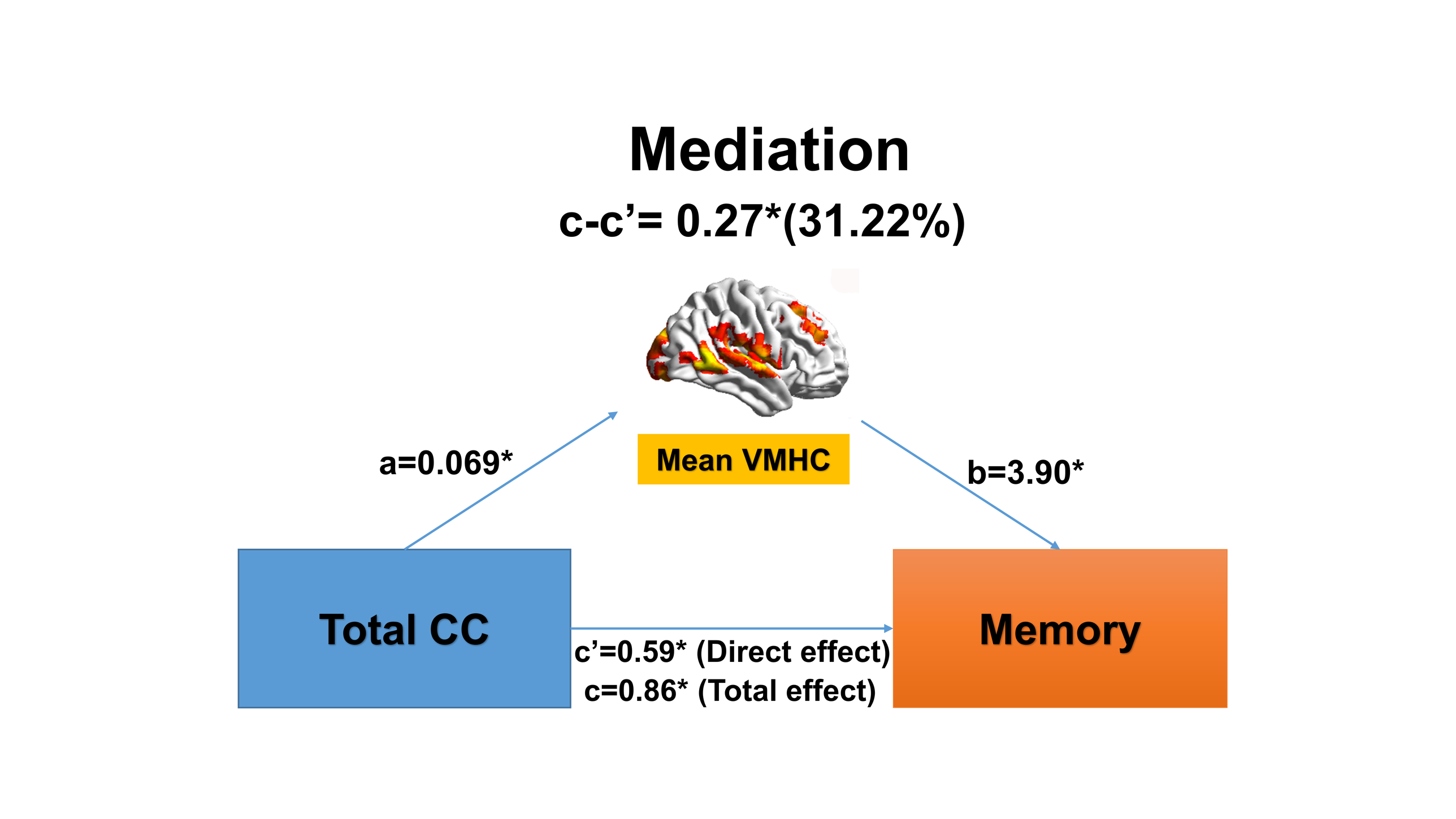


Regions whose inter-hemispheric homotopic functional connectivity (i.e., VMHC) was related to total CC mediated the impact of total CC degeneration on memory deficit in AD and aMCI, after controlling for hippocampal volume. ‘c’ denotes the total effect of CC volume on memory; ‘c’’ denotes the direct effect of CC volume on memory (not through inter-hemispheric homotopic functional connectivity); and ‘c-c’’ denotes the indirect effect (mediated effect, through inter-hemispheric homotopic functional connectivity).’*’ means the significance level of p< 0.05.

**Supplementary Figure** **6**. **Inter-hemispheric homotopic functional connectivity mediated the effect of corpus callosum subregions degeneration on memory in AD and aMCI patients.**


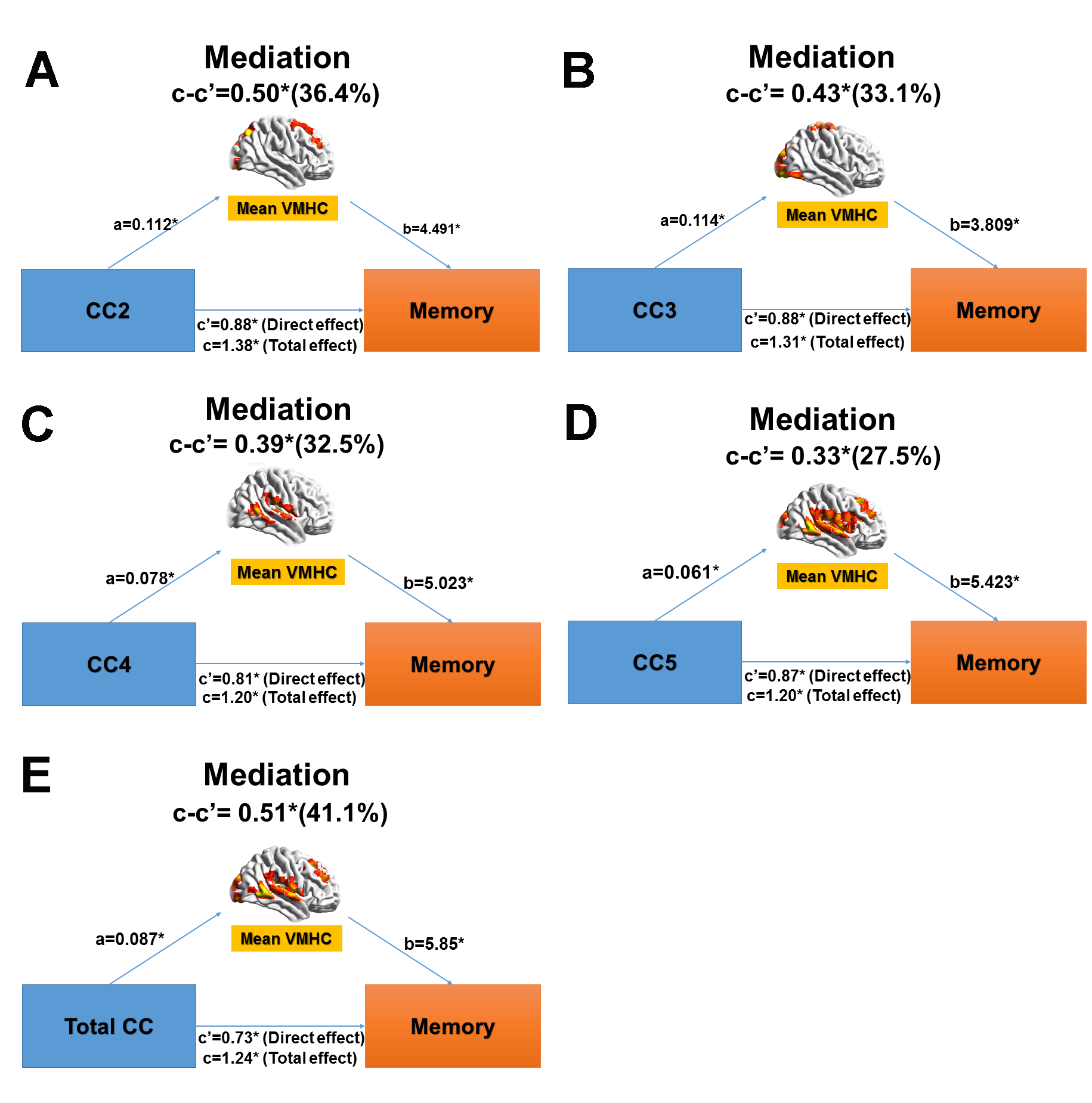


The mean VMHC in the regions show significant correlations with CC subregions (CC2, CC3, CC4, CC5) mediating the effects CC subregions, including CC2 (A), CC3 (B), CC4 (C), CC5 (D) on memory.

‘c’ denotes the total effect of CC volume on memory; ‘c’’ denotes the direct effect of CC volume on memory (not through inter-hemispheric homotopic functional connectivity); and ‘c-c’’ denotes the indirect effect (mediated effect, through inter-hemispheric homotopic functional connectivity).

**References:**

1 Wahlund, L. O. *et al.* A New Rating Scale for Age-Related White Matter Changes Applicable to MRI and CT. *Stroke; a journal of cerebral circulation* **32**, 1318-1322, doi:10.1161/01.str.32.6.1318 (2001).

2 Morris, J. C. The Clinical Dementia Rating (CDR): current version and scoring rules. *Neurology* **43**, 2412-2414 (1993).

3 Folstein, M. F., Folstein, S. E. & McHugh, P. R. "Mini-mental state". A practical method for grading the cognitive state of patients for the clinician. *Journal of psychiatric research* **12**, 189-198 (1975).

4 Nasreddine, Z. S. *et al.* The Montreal Cognitive Assessment, MoCA: a brief screening tool for mild cognitive impairment. *Journal of the American Geriatrics Society* **53**, 695-699, doi:10.1111/j.1532-5415.2005.53221.x (2005).

5 Hilal, S. *et al.* Prevalence of cognitive impairment in Chinese: epidemiology of dementia in Singapore study. *J Neurol Neurosurg Psychiatry* **84**, 686-692, doi:10.1136/jnnp-2012-304080 (2013).

6 Yeo, D. *et al.* Pilot validation of a customized neuropsychological battery in elderly Singaporeans. *Neurol J South East Asia* **2** (1997).

7 Dubois, B., Slachevsky, A., Litvan, I. & Pillon, B. The FAB: a Frontal Assessment Battery at bedside. *Neurology* **55**, 1621-1626 (2000).

8 Porteus, S. D. Recent maze test studies. *The British journal of medical psychology* **32**, 38-43 (1959).

9 Wechsler, D. *WMS-III administration and scoring manual*. ( The Psychological Corporation, Harcourt Brace Jovanovich. , 1997).

10 Lewis, R. F. & Rennick, P. M. *Manual for the Repeatable Cognitive-Perceptual-Motor Battery*. (Axon Publishing Company, 1979).

11 Mack, W. J., Freed, D. M., Williams, B. W. & Henderson, V. W. Boston Naming Test: shortened versions for use in Alzheimer's disease. *Journal of gerontology* **47**, P154-158 (1992).

12 Isaacs, B. & Kennie, A. The Set test as an aid to the detection of dementia in old people. *The British journal of psychiatry : the journal of mental science* **123**, 467-470 (1973).

13 Smith, A. *Symbol Digit Modalities Test*. (Western Psychological Services, 1973).

14 Diller, L., Y, B.-Y. & LJ, G. *Studies in cognition and rehabilitation in hemiplegia*. ( Institute of Rehabilitation Medicine, New York University Medical Center, 1974).

15 Sunderland, T. *et al.* Clock drawing in Alzheimer's disease. A novel measure of dementia severity. *Journal of the American Geriatrics Society* **37**, 725-729 (1989).

16 Wechsler, D. *Wechsler Adult Intelligence Scale-Revised*. (Harcourt Brace Jovanovich, 1981).

17 Sahadevan, S., Tan, N. J., Tan, T. & Tan, S. Cognitive testing of elderly Chinese people in Singapore: influence of education and age on normative scores. *Age and ageing* **26**, 481-486 (1997).

18 McKhann, G. M. *et al.* The diagnosis of dementia due to Alzheimer’s disease: Recommendations from the National Institute on Aging-Alzheimer’s Association workgroups on diagnostic guidelines for Alzheimer's disease. *Alzheimer's & Dementia* **7**, 263-269 (2011).

19 Ségonne, F. *et al.* A hybrid approach to the skull stripping problem in MRI. *Neuroimage* **22**, 1060-1075, doi:10.1016/j.neuroimage.2004.03.032 (2004).

20 Fischl, B. *et al.* Whole brain segmentation: automated labeling of neuroanatomical structures in the human brain. *Neuron* **33**, 341-355 (2002).

21 Fischl, B. *et al.* Automatically parcellating the human cerebral cortex. *Cerebral cortex* **14**, 11-22 (2004).

22 Fischl, B., Liu, A. & Dale, A. M. Automated manifold surgery: constructing geometrically accurate and topologically correct models of the human cerebral cortex. *IEEE transactions on medical imaging* **20**, 70-80, doi:10.1109/42.906426 (2001).

23 Zuo, X. N. *et al.* Growing together and growing apart: regional and sex differences in the lifespan developmental trajectories of functional homotopy. *J Neurosci* **30**, 15034-15043, doi:10.1523/JNEUROSCI.2612-10.2010 (2010).

24 Hoptman, M. J. & Davidson, R. J. How and why do the two cerebral hemispheres interact? *Psychological bulletin* **116**, 195-219 (1994).
